# Sialin-STAT3 axis regulates bone homeostasis in mice

**DOI:** 10.1038/s41413-025-00504-2

**Published:** 2026-02-09

**Authors:** Xiaoyu Li, Lei Hu, Yifan Xu, Xue Wang, Zichen Cao, Ou Jiang, Jiawei Yao, Meijing Liu, Sihan Kong, Jinsong Wang, Xiaogang Wang, Songlin Wang

**Affiliations:** 1 Salivary Gland Disease Center and Beijing Key Laboratory of Tooth Regeneration and Function Reconstruction, Beijing Laboratory of Oral Health and Beijing Stomatological Hospital, Capital Medical University, Beijing, China; 2 The Third Affiliated Hospital of Southern Medical University, Southern Medical University, Guangzhou, Guangdong China; 3 Department of Biochemistry and Molecular Biology, Capital Medical University School of Basic Medicine, Beijing, China; 4 Laboratory of Oral Homeostatic Medicine, School of Medicine and SUSTech Homeostatic Medicine Institute (SHMI), Southern University of Science and Technology, Shenzhen, China

**Keywords:** Bone, Bone quality and biomechanics

## Abstract

Mitochondrial regulation in mesenchymal stem cells (MSCs) serves as a critical determinant of bone formation and skeletal homeostasis. While dietary nitrate and its transporter Sialin are implicated in systemic homeostasis, their specific roles in MSCs' function remain unclear. Here, we demonstrate that Sialin deficiency impairs MSCs' function and disrupts bone homeostasis. Gain- and loss-of-function studies reveal that Sialin localizes to the mitochondrial membrane and promotes osteogenic differentiation by maintaining mitochondrial bioenergetic integrity. Mechanistically, Sialin recruits pSTAT3^S727^ to mitochondria, forming a functional complex that activates mitochondrial bioenergy and stabilizes bone remodeling. Notably, dietary nitrate restores Sialin expression in aged mice, thereby enhancing MSCs' function and preventing osteoporosis. Our findings identify a nutrient-responsive signaling axis—nitrate-Sialin-pSTAT3^S727^—that promotes osteogenic differentiation via mitochondrial homeostasis, offering a potential therapeutic strategy for age-related osteoporosis.

## Introduction

The maintenance of skeletal homeostasis—a dynamic equilibrium between osteogenic formation and resorptive processes—represents a fundamental challenge in aging organisms. As the structural foundation of the musculoskeletal system, bone undergoes continuous remodeling orchestrated by mesenchymal stem cells (MSCs) that balance self-renewal and lineage commitment.^1–3^ Age-related dysregulation of this process manifests as osteopenia and osteoporosis, conditions characterized by compromised bone quality and elevated fracture risk.^4^ Despite clinical availability of anti-resorptive drugs and anabolic agents, current strategies fail to fully restore bone quality due to off-target effects and incomplete targeting of MSCs differentiation pathways.^5^ This therapeutic gap underscores the need to identify novel targets that fundamentally recalibrate MSCs' metabolism to restore skeletal homeostasis.^6,7^

Mitochondrial plasticity—the organelle’s capacity to adapt energy metabolism to cellular demands—has emerged as a central regulator of MSCs' fate determination.^8–10^ Beyond their canonical role in ATP synthesis through oxidative phosphorylation,^11,12^ mitochondria integrate metabolic intermediates with epigenetic modifiers to direct lineage specification.^13,14^ Recent studies highlight mitochondrial membrane dynamics and electron transport chain (ETC) efficiency as critical determinants of osteogenic commitment,^15^ while age-related mitochondrial dysfunction accelerates MSCs senescence and skeletal decline.^16,17^ However, the molecular sensors linking extracellular cues to mitochondrial adaptation in MSCs remain elusive.

Intriguingly, dietary nitrate—a ubiquitous nutrient in plant-rich diets—has emerged as a pleiotropic regulator of tissue homeostasis.^18,19^ Beyond cardiovascular benefits mediated through the nitrate-nitrite-NO pathway, nitrate exhibits direct cellular effects via solute carrier family 17 member 5 (Sialin/*SLC17A5*)—a nitrate transporter that facilitates nitrate influx and regulates organelle cross-talk.^20,21^ Preclinical studies have demonstrated that nitrate preserves gastrointestinal homeostasis by modulating the gut microbiota,^22^ attenuates radiation-induced salivary gland damage by suppressing apoptotic pathways,^23^ and prevents bone loss associated with estrogen deficiency.^24^ Notably, nitrate enhances mitochondrial respiratory efficiency in human skeletal muscle,^25^ preserves cardiac mitochondrial integrity during ischemia-reperfusion injury,^26^ and alleviates salivary gland injury by maintaining mitochondrial homeostasis and reducing ROS via the nitrate–Sialin axis.^27^ Nevertheless, critical knowledge gaps persist at the intersection of nutrient signaling and stem cell biology: whether Sialin directly governs MSCs' osteogenesis through mitochondrial metabolic control, how nutrient-sensing pathways interface with transcriptional regulators of mitochondrial function, and whether age-related nitrate deficiency contributes to skeletal aging through stem cell metabolic paralysis.

In this study, we identify Sialin as a mitochondrial-nutrient integrator that translates dietary nitrate availability into osteogenic commitment through STAT3-mediated bioenergetic regulation. By integrating genetic perturbation and pharmacological modulation, we demonstrate that mitochondrial-localized Sialin directly interacts with pSTAT3^S727^ to form a functional complex essential for MSCs differentiation. This nutrient-responsive complex mediates nitrate-driven metabolic adaptation, effectively reversing age-associated MSCs dysfunction and restoring skeletal homeostasis. Our findings establish Sialin as a pivotal metabolic regulator governing MSCs' osteogenesis and underscore the therapeutic potential of targeting the nitrate–Sialin axis to combat age-related skeletal deterioration.

## Results

### Sialin promotes MSCs' osteogenic differentiation and is essential for maintaining bone mass in vivo

**Fig. 1:**
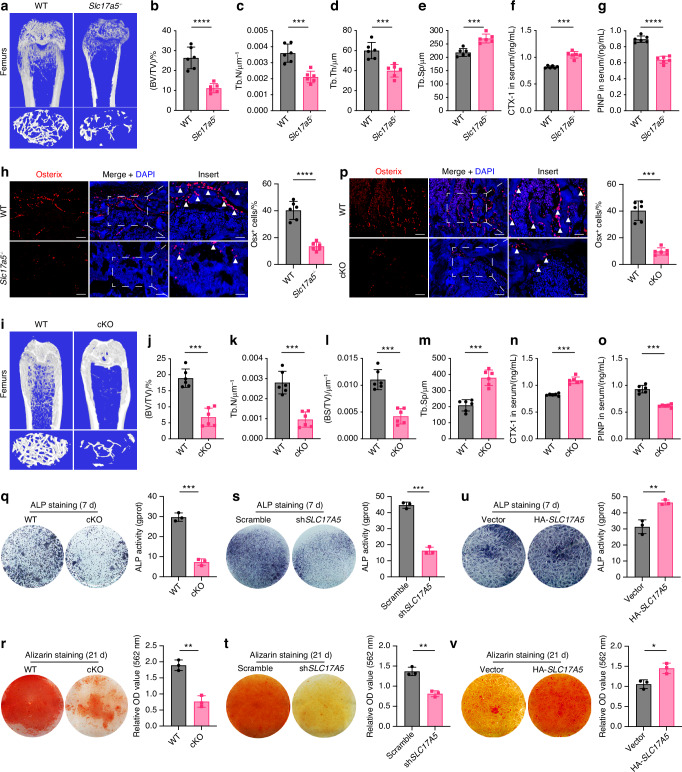
Sialin promotes osteogenic differentiation of mesenchymal stem cells and maintains bone homeostasis. **a** Representative micro-CT images of femurs from WT and *Slc17a5*^-/-^ mice. Upper scale bars, 1 mm. Lower scale bars, 200 μm. Quantification of bone volume/tissue volume (BV/TV, **b**), trabecular number (Tb.N, **c**), trabecular thickness (Tb.Th, **d**), and trabecular separation (Tb.Sp, **e**) in WT and *Slc17a5*^-/-^ mice. Serum levels of CTX-1 (**f**) and PINP (**g**) in WT and *Slc17a5*^*-/-*^ mice. **h** Immunofluorescence (IF) staining images (left) and quantification (right) of osterix (Osx) in femoral sections from WT and *Slc17a5*^-/-^ mice. Scale bars, 50 μm. Arrowheads indicate Osx-positive osteoblasts. **i** Representative micro-CT images of femurs from WT and cKO mice. Upper scale bars, 1 mm. Lower scale bars, 200 μm. Quantification of BV/TV (**j**), Tb. N (**k**), bone surface/tissue volume (BS/TV, **l**), and Tb.Sp (**m**) in WT and cKO mice. Serum CTX-1 (**n**) and PINP (**o**) levels in WT and cKO mice. **p** IF staining images (left) and quantification (right) of Osx in femoral sections from WT and cKO mice. Scale bars, 50 μm. Arrowheads indicate Osx-positive osteoblasts. ALP staining and activity (**q**), and ARS staining with quantification of mineralized nodules (**r**) in murine MSCs (mMSCs) from 8-week-old WT and cKO male mice. ALP staining and activity (**s**), and ARS staining and mineralization (**t**) in control and *SLC17A5* knockdown (sh*SLC17A5*) human MSCs (hMSCs). ALP staining and activity (**u**), and ARS staining and mineralization (**v**) in control and Sialin-overexpressing (HA-*SLC17A5*) hMSCs. Data are presented as the mean ± SD, *n* = 3, except for **a**–**p** (*n* = 6). *P < 0.05; **P < 0.01; ***P < 0.001

We constructed *Slc17a5*^*−/−*^ mice and found that Sialin plays a key role in maintaining bone homeostasis in mice. Western blot analysis indicated successful deletion of *Slc17a5* (Fig. S1a). Micro-computed tomography (μCT) images of the femur are shown in Fig. 1a. The results of bone histomorphometry indexes were obtained through quantitative analysis. The bone volume fraction (BV/TV), trabecular thickness (Tb.Th), and number of trabeculae (Tb.N) of the femur in *Slc17a5*^*−/−*^ mice were lower than those in the wild-type (WT) control group (Fig. 1b–d), while the trabecular separation (Tb.Sp) was higher in *Slc17a5*^*−/−*^ mice than that in the control group (Fig. 1e), indicating that *Slc17a5*^*−/−*^ mice had relatively low bone mass. At the femoral mid-diaphysis, cortical bone volume (Ct.BV), cortical thickness (Ct.Th), and total trabecular area (Tt.Ar) were markedly decreased in *Slc17a5*^*−/−*^ mice, consistent with the trabecular phenotype (Fig. S1d–f). The bone resorption marker collagen type I cross-linked C-telopeptide (CTX-1) were significantly elevated in *Slc17a5*^*−/−*^ mice compared to that in WT (Fig. 1f). The bone formation marker procollagen type I N-propeptide (P1NP) was significantly decreased in *Slc17a5*^*−/−*^ mice compared to that in WT (Fig. 1g). These results suggest that Sialin loss impairs bone homeostasis by altering bone remodeling. Immunofluorescence (IF) staining of bone sections revealed lower number of Osterix (Osx)-positive osteoblasts in *Slc17a5*^*−/−*^ mice than in the control mice (Fig. 1h). We further performed tartrate-resistant acid phosphatase (TRAP) staining on femoral sections and found that *Slc17a5*^*−/−*^ mice exhibited increased osteoclast formation compared to control mice (Fig. S1b).

To verify the effect of *Slc17a5* expression in MSCs on bone formation, we generated *Slc17a5* flox (*Slc17a5*^*fl/fl*^) mice using CRISPR/Cas9 technique and bred *Slc17a5*^*fl/fl*^ mice with *Prx1-Cre* transgenic mice to obtain conditional homozygous *Slc17a5* knockout (KO), *Prx1-Cre; Slc17a5*^*fl/fl*^ (cKO) mice. Western blot analysis indicated successful deletion of *Slc17a5* in stromal cells isolated from long bones of *Slc17a5* knockout mice (Fig. 1s). Micro-CT images of the femur are shown in Fig. 1i. The BV/TV, Tb.N, and bone surface fraction (BS/BV) of the femur in cKO mice were lower than those in the control group (Fig. 1j–l), while the Tb.Sp was in cKO mice higher than that in the control group (Fig. 1m), indicating that *SLC17A5* may regulate osteogenic differentiation in MSCs and bone formation in mice. Similarly, in cKO mice, cortical bone indices were significantly reduced compared with their littermate WT controls. Ct.BV, Ct.Th, and Tt.Ar all showed decreases, again paralleling the trabecular findings (Fig. S1g–i). CTX-1 level was significantly elevated (Fig. 1n), while P1NP level was significantly decreased (Fig. 1o) in cKO mice compared to those in the WT. IF staining of bone sections revealed a lower number of Osx^+^ osteoblasts in cKO mice than in the WT (Fig. 1p). TRAP staining of femoral sections indicated elevated numbers of TRAP^+^ osteoclasts in cKO mice relative to controls (Fig. S1c). To verify whether Sialin regulates bone homeostasis by modulating the function of MSCs, we isolated MSCs from cKO and control mice. Significant reductions in alkaline phosphatase (ALP) staining intensity and activity, and alizarin red S (ARS) staining intensity and generation of mineralized nodules were noticed in MSCs of cKO mice on days 7 and 21 of differentiation, respectively (Fig. 1q, r). The mRNA expression of the key osteogenesis-related genes, such as *Alpl*, *Runx2*, and *Spp1* (Fig. S1j–l), and expression of osteogenesis-related proteins, such as COL-I, RUNX2, and OCN (Fig. S1m), were also significantly downregulated, confirming the effect of Sialin on the osteogenic potential of murine MSCs.

To further clarify whether Sialin affects cellular osteogenic differentiation, we knocked down *SLC17A5(Slc17a5)* in human MSCs (hMSCs) and murine MSCs (mMSCs) using short hairpin (sh) RNA sequences. Sialin expression was significantly decreased (Figs. 1v and S2a). After 7 days of differentiation, the ALP staining intensity of *SLC17A5(Slc17a5)* knockdown hMSCs and mMSCs was markedly reduced compared to their respective controls, with a similar trend observed in the ALP activity assays (Figs. 1t and S2b). After 21 days of differentiation, ARS staining and quantitative analysis showed that the formation of mineralized nodules was significantly reduced in *SLC17A5(Slc17a5)* knockdown hMSCs and mMSCs (Figs. 1u and S2c). In hMSCs, the mRNA expression of osteogenesis-related genes, including *ALPL*, *COL1A1*, *BGLAP*, *RUNX2*, and *SP7* (Fig. S1n–r), and the protein expression of COL-I, RUNX2, and OCN were markedly decreased (Fig. S1s).

Next, we overexpressed *SLC17A5(Slc17a5)* in hMSCs and mMSCs, which led to a significant increase in Sialin protein levels (Figs. 1y and S2d). After 7 days of differentiation, the ALP staining intensities of *SLC17A5(Slc17a5)*-overexpressed hMSCs and mMSCs were markedly higher than those in control cells, consistent with the observed increase in ALP activity (Figs. 1w and S2e). After 21 days, ARS staining showed enhanced mineral deposition in both *SLC17A5(Slc17a5)*-overexpressed hMSCs and mMSCs, with a significant increase in mineralized nodule formation (Fig. 1x and S2f). In hMSCs, the mRNA expression of osteogenic genes, including *ALPL*, *COL1A1*, *SPP1*, *RUNX2*, and *SP7* (Fig. S1t–x), as well as the protein expression levels of COL-I, RUNX2, and OCN, were significantly upregulated (Fig. S1y). These findings indicate that Sialin promotes osteogenic differentiation in MSCs.

### Mitochondrial localization of Sialin supports MSCs' osteogenesis by regulating mitochondrial function

**Fig. 2:**
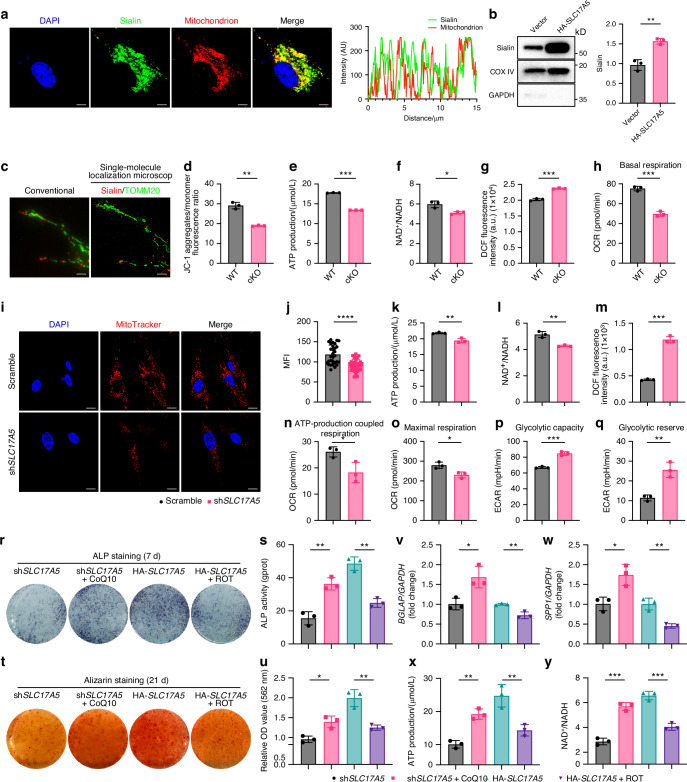
Sialin localizes in mitochondria and regulates osteogenic differentiation of MSCs via mitochondrial function. **a** IF staining images showing colocalization of Sialin (green) with mitochondria (MitoTracker, red) in hMSCs. Nuclei were counterstained with DAPI (blue). Scale bars, 10 μm. **b** Immunoblot analysis of Sialin expression in mitochondrial fractions from control and Sialin-overexpressing (HA-*SLC17A5*) hMSCs. **c** Single-molecule localization microscopy showing Sialin (red) colocalized with TOMM20 (green) in hMSCs. Scale bars, 1 μm. Quantification of JC-1 polymer/monomer ratio (**d**), ATP level (**e**), NAD^+^/NADH ratio (**f**), ROS production (**g**), and basal respiration (**h**) in mMSCs from 8-week-old WT and cKO male mice. IF staining images (**i**) and quantitation (**j**) of MitoTracker fluorescence in control and sh*SLC17A5* hMSCs. Scale bars, 25 μm. ATP level (**k**), NAD^+^/NADH ratio (**l**), ROS level (**m**), ATP production (**n**), maximal respiration (**o**), glycolytic capacity (**p**), and glycolytic reserve (**q**) in control and sh*SLC17A5* hMSCs. ALP staining and activity (**r**, **s**), and ARS staining with quantification of mineralized nodules (**t**, **u**) in sh*SLC17A5* hMSCs treated with CoQ10 and Sialin-overexpressing hMSCs treated with ROT. RT-qPCR analysis of *BGLAP* (**v**) and *SPP1* (**w**) mRNA expression in sh*SLC17A5* hMSCs treated with CoQ10 and Sialin-overexpressing hMSCs treated with ROT. ATP production (**x**) and NAD^+^/NADH ratio (**y**) in sh*SLC17A5* hMSCs treated with CoQ10 and Sialin-overexpressing hMSCs treated with ROT. Data are presented as the mean ± SD, *n* = 3, except for **j** and **s** (*n* = 30). *P < 0.05; **P < 0.01; ***P < 0.001; ****P < 0.000 1

Mitochondria, as the energy center of all eukaryotic cells, play a crucial role in regulating the function of MSCs. Colocalization studies of Sialin and the mitochondrial marker, Mito Tracker, revealed that Sialin was localized to mitochondria in hMSCs (Fig. 2a), and that Sialin expression was significantly high in the mitochondrial fraction of *SLC17A5*-overexpressed hMSCs (Fig. 2b). To minimize crosstalk and perform an accurate colocalization analysis, we used two-color single-molecule localization microscopy to reveal the colocalization of Sialin with mitochondrial membrane TOMM20 in hMSCs (Fig. 2c).

Subsequently, Mito Tracker and JC-1 staining demonstrated that the fluorescence intensity (Fig. S3a) and JC-1 polymer/monomer ratio (Fig. 2d) were reduced in cKO mMSCs, suggesting that the mitochondrial membrane potential (MMP) decreased. The ATP level (Fig. 2e) and NAD^+^/NADH ratio (Fig. 2f) were significantly lower in the mitochondria of cKO mMSCs than those in the WT; however, the extent of ROS production increased (Fig. 2g). In addition, the oxygen consumption rate (OCR) of cKO mMSCs was assessed, and non-mitochondrial oxygen consumption and basal respiration were found to be significantly decreased (Figs. S3b and 2h).

Measurement of MMP using MitoTracker and JC-1 staining revealed a reduction in fluorescence intensity in *SLC17A5* knockdown hMSCs compared to control cells (Fig. 2i, j), indicating impaired MMP. Consistently, intracellular ATP levels (Fig. 2k) and the NAD^+^/NADH ratio (Fig. 2l) were markedly decreased, whereas ROS levels (Fig. 2m) were significantly elevated in *SLC17A5* knockdown hMSCs. Mitochondrial stress testing via OCR and extracellular acidification rate (ECAR) analysis revealed significantly reduced ATP production in *SLC17A5* knockdown hMSCs compared with controls (Fig. 2n), suggesting diminished mitochondrial capacity to meet basal energy demands. Addition of the uncoupler carbonyl cyanide p-trifluoro-methoxyphenyl hydrazone (FCCP) further revealed that maximal respiration was significantly impaired in *SLC17A5* knockdown hMSCs (Fig. 2o), indicating reduced bioenergetic responsiveness under stress conditions. Similarly, spare respiratory capacity was markedly decreased (Fig. S3c), further supporting a loss of metabolic flexibility. In mMSCs, basal respiration, ATP production, and maximal respiration were likewise significantly decreased following *Slc17a5* knockdown (Fig. S3d–g). Interestingly, *SLC17A5* knockdown hMSCs displayed an increase in both basal glycolytic capacity (Fig. 2p) and maximal glycolytic response (Fig. 2q), indicating a compensatory metabolic shift toward glycolysis.

Enhanced fluorescence intensity of MitoTracker staining (Fig. S3h, i) indicated that *SLC17A5* overexpression increased MMP in hMSCs. Consistently, *SLC17A5* overexpression significantly elevated intracellular ATP levels (Fig. S3j) and the NAD^+^/NADH ratio (Fig. S3k), while reducing ROS production (Fig. S3l). Basal respiration (Fig. S3m) and ATP production (Fig. S3n) were both significantly higher in *SLC17A5*-overexpressed hMSCs compared to control cells, suggesting an enhanced capacity for mitochondrial ATP synthesis to meet cellular energy demands. Upon FCCP treatment, maximal respiration was significantly increased in *SLC17A5*-overexpressed hMSCs (Fig. S3o), indicating improved bioenergetic responsiveness under stress conditions. Similar enhancements in basal respiration, ATP production, and maximal respiration were observed in *Slc17a5*-overexpressed mMSCs (Fig. S3p–s). Interestingly, glycolytic capacity (Fig. S3t) and glycolytic reserve capacity (Fig. S3u) were both reduced in *SLC17A5*-overexpressed hMSCs compared to controls, suggesting a metabolic shift favoring mitochondrial oxidative phosphorylation over glycolysis.

To determine whether Sialin influences the osteogenic differentiation potential of MSCs through regulation of mitochondrial homeostasis, we modulated mitochondrial electron transport chain (ETC) activity by pharmacological means: *SLC17A5* knockdown hMSCs were treated with Coenzyme Q10 (CoQ10) to enhance ETC activity, while *SLC17A5*-overexpressed hMSCs were treated with rotenone (ROT) to inhibit it. After 7 days of differentiation, CoQ10 treatment enhanced ALP staining in *SLC17A5* knockdown hMSCs, whereas ROT treatment led to a marked reduction in ALP staining intensity in *SLC17A5*-overexpressed hMSCs, with consistent changes observed in ALP activity assays (Fig. 2r, s). After 21 days, ARS staining showed increased mineralization and nodule formation in CoQ10-treated *SLC17A5* knockdown hMSCs, whereas ROT-treated *SLC17A5*-overexpressed hMSCs displayed reduced mineral deposition (Fig. 2t, u). In parallel, qPCR analysis revealed significant upregulation of *BGLAP* and *SPP1* in *SLC17A5* knockdown hMSCs treated with CoQ10, and downregulation of these genes in *SLC17A5*-overexpressed hMSCs following ROT treatment (Fig. 2v, w). CoQ10 activation also restored ATP levels and the NAD^+^/NADH ratio in *SLC17A5* knockdown hMSCs, while ROT treatment impaired both parameters in *SLC17A5*-overexpressed hMSCs (Fig. 2x, y). Collectively, these findings demonstrate that Sialin promotes osteogenic differentiation of MSCs by supporting mitochondrial function through regulation of ETC activity.

### Mitochondrial-targeted Sialin overexpression enhances metabolic and functional homeostasis in MSCs

**Fig. 3:**
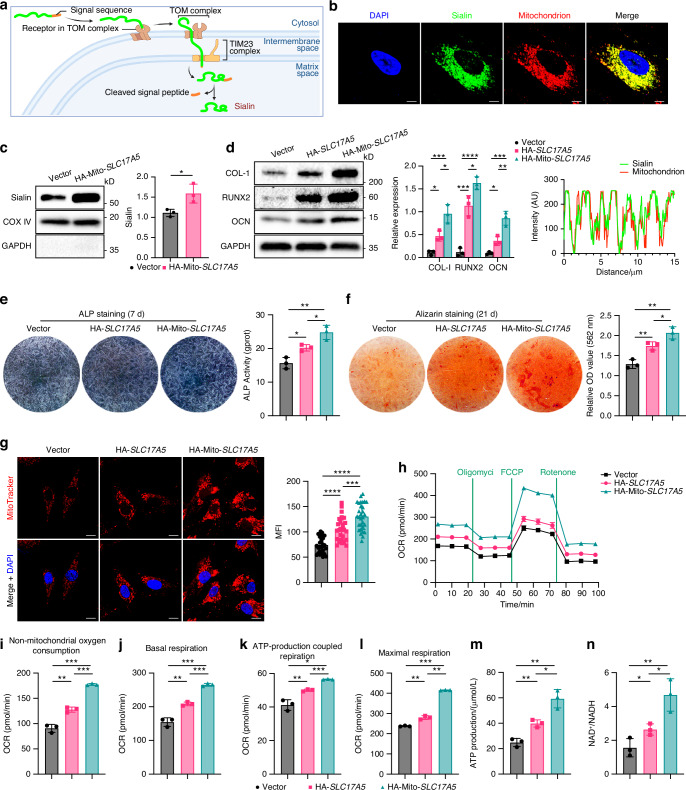
Mitochondria-targeted Sialin more effectively maintains MSCs homeostasis. **a** Schematic diagram of the construct for mitochondria-targeted overexpression of Sialin. **b** IF staining images showing colocalization of mitochondria-targeted Sialin (green) with mitochondria (MitoTracker, red) hMSCs. Nuclei were stained with DAPI (blue). Scale bars, 10 μm. **c** Immunoblot analysis of Sialin expression in mitochondrial fractions from control and mitochondria-targeted Sialin-overexpressing (HA-Mito-*SLC17A5*) hMSCs. **d** Immunoblot analysis of COL-I, RUNX2, Sialin, and OCN in control, Sialin-overexpressing (HA-*SLC17A5*), and mitochondria-targeted Sialin-overexpressing hMSCs. ALP staining and activity (**e**), and ARS staining with quantification of mineralized nodules (**f**) in control, Sialin-overexpressing, and mitochondria-targeted Sialin-overexpressing hMSCs. **g** IF staining images and quantitation of MitoTracker fluorescence intensity in control, Sialin-overexpressing, and mitochondria-targeted Sialin-overexpressing hMSCs. Scale bars, 25 μm. **h** OCR profiles of control, Sialin-overexpressing, and mitochondria-targeted Sialin-overexpressing hMSCs. Non-mitochondrial oxygen consumption (**i**), basal respiration (**j**), ATP production (**k**), and maximal respiration (**l**) in control, Sialin-overexpressing, and mitochondria-targeted Sialin-overexpressing hMSCs. ATP levels (**m**) and NAD^+^/NADH ratio (**n**) in control, Sialin-overexpressing, and mitochondria-targeted Sialin-overexpressing hMSCs. Data are presented as the mean ± SD, *n* = 3, except for g (*n* = 30). *P < 0.05; **P < 0.01; ***P < 0.001

To specifically target Sialin to the mitochondria of hMSCs, we fused the mitochondrial targeting sequence derived from the lead peptide of cytochrome c oxidase subunit 8 (COX8) to the N-terminus of HA-tagged *SLC17A5*, generating a mitochondria-targeted Sialin construct (Fig. 3a, b). This mitochondrial-targeted overexpression significantly elevated Sialin protein levels in hMSCs (Figs. 3c, S3v). Following 7 days of osteogenic induction, hMSCs expressing mitochondria-targeted Sialin exhibited denser ALP staining than both control and conventional *SLC17A5*-overexpressed hMSCs (Fig. 3e). After 21 days, these cells also showed enhanced ARS staining and increased mineralized nodule formation (Fig. 3f). Consistently, the protein levels of osteogenic markers COL-I, RUNX2, Sialin, and OCN were upregulated in hMSCs with mitochondria-targeted *SLC17A5* overexpression compared to both control and non-targeted *SLC17A5*-overexpressing cells (Fig. 3d).

MitoTracker fluorescence intensity was more markedly increased in hMSCs with mitochondria-targeted *SLC17A5* overexpression than in those with non-targeted *SLC17A5* overexpression (Fig. 3g), indicating a greater enhancement of MMP. We subsequently assessed the OCR in control, *SLC17A5*-overexpressed, and mitochondria-targeted *SLC17A5*-overexpressed hMSCs (Fig. 3h). Mitochondria-targeted *SLC17A5* overexpression significantly increased basal respiration (Fig. 3j), non-mitochondrial oxygen consumption (Fig. 3i), and ATP production (Fig. 3k), even after ROT/antimycin A treatment. Following FCCP administration, maximum respiration was highest in hMSCs overexpressing mitochondria-targeted *SLC17A5* (Fig. 3l), indicating an improved capacity to respond to elevated energy demand or metabolic stress. In addition, mitochondrial targeting led to a further increase in ATP levels (Fig. 3m) and the NAD^+^/NADH ratio (Fig. 3n) compared to both control and non-targeted *SLC17A5*-overexpressed hMSCs. Collectively, these results confirm that mitochondria-targeted *SLC17A5* overexpression more effectively enhances mitochondrial homeostasis and promotes osteogenic differentiation of MSCs.

### Sialin interacts with pSTAT3^S727^ and facilitates its mitochondrial translocation

**Fig. 4:**
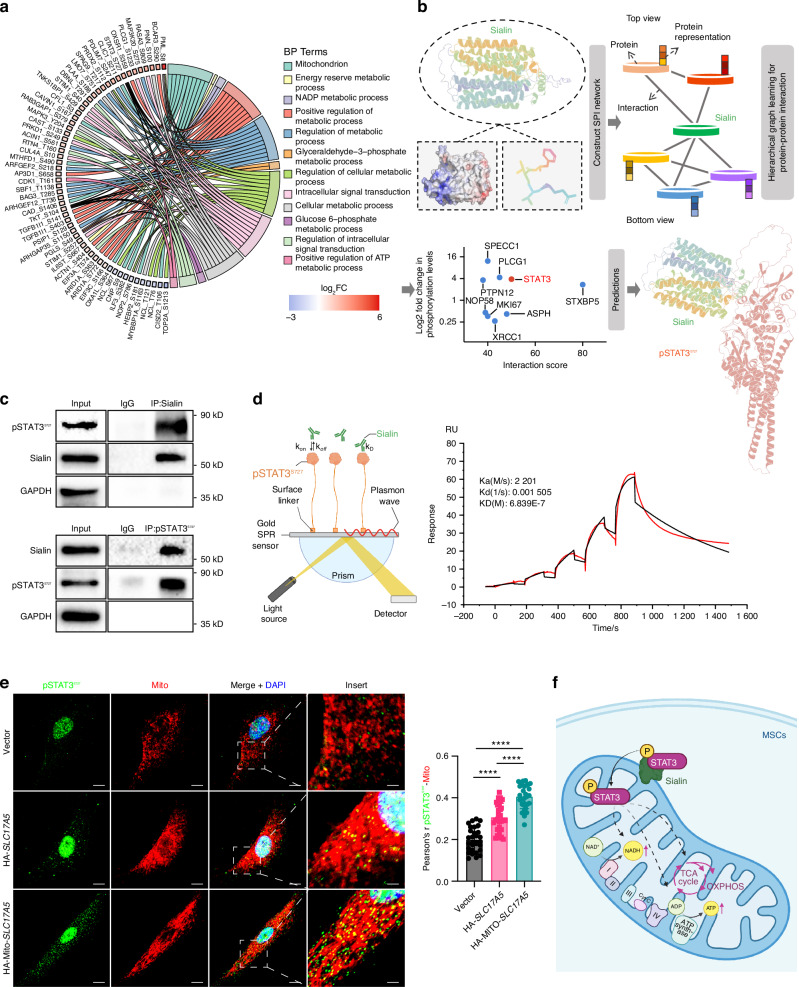
Sialin interacts with pSTAT3^S727^ and promotes its mitochondrial translocation in MSCs. **a** Gene Ontology biological process (GO-BP) enrichment analysis of the differentially phosphorylated proteins. **b** Dual-view hierarchical graph learning model predicts the interaction between Sialin and candidate proteins. **c** Co-immunoprecipitation (Co-IP) showing the interaction between Sialin and pSTAT3^S727^ in hMSCs. **d** Surface plasmon resonance (SPR) analysis confirming the direct binding between Sialin and pSTAT3^S727^ in hMSCs (KD = 683 nmol/L). **e** IF staining images and quantitation of pSTAT3^S727^ colocalization with mitochondria by Pearson’s correlation coefficient in control, Sialin-overexpressing (HA-*SLC17A5*), and mitochondria-targeted Sialin-overexpressing (HA-Mito-*SLC17A5*) hMSCs. Scale bars, 10 μm. **f** A schematic model: Sialin binds pSTAT3^S727^, promotes its translocation to mitochondria, and thereby activates mitochondrial function in hMSCs. Data are presented as the mean ± SD, *n* = 3, except for **f** (*n* = 30). ****P < 0.000 1

To further explore the molecular mechanism by which Sialin regulates mitochondrial function, we extracted mitochondrial proteins from control and *SLC17A5* knockdown hMSCs for phosphoproteomic analysis. PCA (Fig. S4a), combined with heatmap analysis, indicated significant differences in phosphorylation profiles between the two groups (Fig. S4b). A volcano plot of all the proteins in the differential phosphorylation analysis was generated, with red representing 116 significantly upregulated phosphoproteins and blue representing 226 significantly downregulated ones. Subsequently, the differentially phosphorylated proteins were subjected to gene ontology (GO) biological process (BP) enrichment (Fig. 4a), Reactome pathway (Fig. S4c), and WikiPathways analysis (Fig. S4d), which revealed that most of these proteins were involved in mitochondrial functions, various metabolic pathways, and signal transduction processes. These results support the critical role of Sialin in maintaining MSCs homeostasis by regulating mitochondrial function and cellular metabolism.

Recently, a two-view layer hierarchical map learning model called HIGH-PPI was reported, which applies dual networks to represent protein structure and interaction interface structure, and achieves high accuracy and robustness in protein-protein interactions (PPIs) prediction.^28^ This model also enables identification of key binding sites and provides mechanistic interpretability. We applied the HIGH-PPI model to predict potential Sialin interactors among the differentially phosphorylated proteins and integrated these predictions with our phosphoproteomic data into a scatter plot (Fig. 4b), where the x-axis represents the HIGH-PPI–derived interaction score and the y-axis represents the change in phosphorylation level. This analysis highlighted pSTAT3^S727^ as the highest-scoring differentially phosphorylated protein associated with mitochondrial regulation, while other candidates exhibited low predicted binding capacity or lacked detectable interaction regions. Therefore, we chose pSTAT3^S727^ as the focus of subsequent studies. We confirmed that Sialin interacted with pSTAT3^S727^ in hMSCs, using classical co-immunoprecipitation (CoIP) (Fig. 4c). To clarify the binding strength of Sialin and pSTAT3^S727^, we used surface plasmon resonance (SPR) and found the binding constant (*K*_a_) of 2.201E + 3, dissociation constant (*K*_d_) of 1.505E-3, and affinity constant (KD) of 6.839E-7, which indicated strong binding (Fig. 4d). pSTAT3^S727^ affects mitochondrial function by enhancing the activity of the electron transport chain and increasing ATP production, and is critical for bone homeostasis by regulating osteogenesis.^29–31^ Furthermore, we found that the mitochondria-targeted *SLC17A5* overexpression significantly promoted pSTAT3^S727^ expression in the mitochondria of hMSCs (Fig. 4e). In summary, these findings indicate that Sialin can interact with pSTAT3^S727^ and positively regulate the mitochondrial translocation of pSTAT3^S727^ in hMSCs to activate mitochondrial function (Fig. 4f).

### Sialin-pSTAT3^S727^ axis controls MSCs osteogenesis and bone homeostasis via mitochondrial regulation

**Fig. 5:**
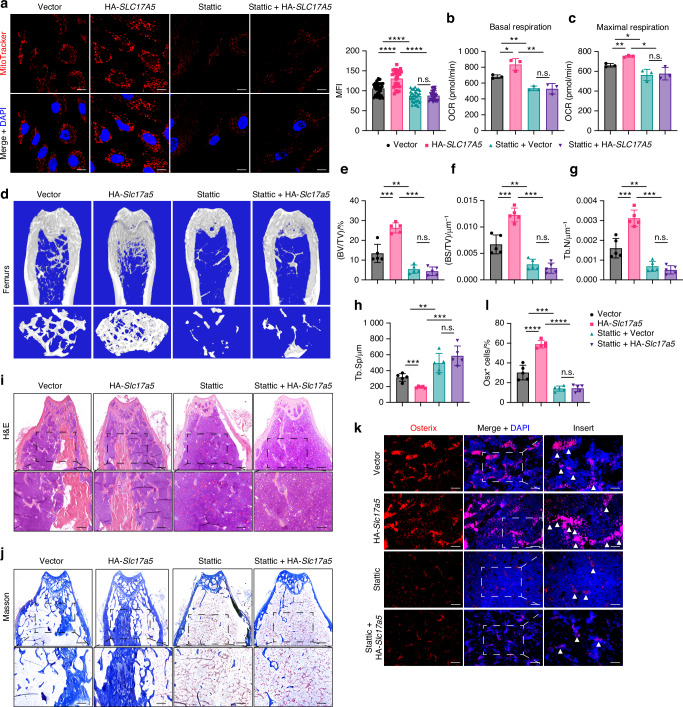
Inhibition of STAT3 phosphorylation abolishes the Sialin-induced enhancement of MSCs function and bone homeostasis. **a** IF staining images and quantitation of MitoTracker intensity in control and Sialin-overexpressing (HA-*SLC17A5*) hMSCs treated with the STAT3 phosphorylation inhibitor Stattic. Scale bars, 25 μm. Basal respiration (**b**) and maximal respiration (**c**) in control and Sialin-overexpressing hMSCs after Stattic treatment. **d** Representative micro-CT images of femurs. Upper scale bars, 1 mm. Lower scale bars, 200 μm. Quantification of BV/TV (**e**), BS/BV (**f**), Tb.N (**g**), and Tb.Sp (**h**) in femurs of control and Sialin-overexpressing mice treated with Stattic. H&E (**i**) and Masson’s trichrome staining (**j**) of femoral sections from control and Sialin-overexpressing mice treated with Stattic. Upper scale bars, 1 mm. Lower scale bars, 200 μm. IF staining images (**k**) and quantification (**l**) of Osx in femoral sections from control and Sialin-overexpressing mice treated with Stattic. Scale bars, 50 μm. Arrowheads indicate Osx-positive osteoblasts. Data are presented as the mean ± SD, *n* = 3, except for a (*n* = 30), **d**–**l** (*n* = 5). *P < 0.05; **P < 0.01; ***P < 0.001; ****P < 0.000 1; n.s., no significance

STAT3 has been considered as a potential target in several diseases, including cancer, immune disorders, and bone metabolism and homeostasis.^32^ Therefore, we wondered whether Sialin is a suitable pharmacological target for regulating bone homeostasis through pSTAT3. The classical STAT3 inhibitor, Stattic, inhibits the phosphorylation and activity of STAT3. To clarify the essential role of Sialin in regulating mitochondrial function, we inhibited STAT3 phosphorylation using Stattic and observed that MMP in hMSCs was significantly reduced and could no longer be enhanced by *SLC17A5* overexpression (Fig. 5a). In line with this, cellular NAD^+^/NADH ratio and ATP levels were markedly decreased upon STAT3 inhibition, and *SLC17A5* overexpression failed to restore them (Fig. S5a, b). Moreover, *SLC17A5(Slc17a5)* overexpression did not increase either basal or maximal respiration in Stattic-treated hMSCs and mMSCs, suggesting that pSTAT3^S727^ is required for Sialin-mediated enhancement of mitochondrial responsiveness under increased energy demand or stress (Figs. 5b, c and S5c, d).

Consistently, after 7 and 21 days of osteogenic induction, ALP staining intensity and activity, as well as ARS staining and mineralized nodule formation, were all reduced in Stattic-treated hMSCs and mMSCs compared to untreated controls (Fig. S5e–h). Importantly, *SLC17A5(Slc17a5)* overexpression did not rescue these osteogenic defects in the presence of Stattic (Fig. S5e–h). Nuclear counts using DAPI staining revealed comparable cell numbers between Stattic-treated and control groups, indicating that the observed reductions in ALP and mineralization were not due to cell loss (Fig. S5i). To further assess whether constitutive activation of STAT3 could compensate for Sialin deficiency, we expressed a phosphomimetic STAT3-S727D mutant in *SLC17A5* knockdown hMSCs. STAT3-S727D partially restored ALP activity and osteogenic gene expression compared with *SLC17A5* knockdown hMSCs transfected with empty vector, but the rescue remained incomplete relative to controls (Fig. S6a, b). To further explore the in vivo effects of pharmacological inhibition of STAT3 activity, 8-week-old mice were intraperitoneally injected with 10 mg/kg Stattic thrice per week for 4 weeks. Micro-CT analysis indicated that the trabecular bone was reduced by Stattic treatment, indicated by decreased BV/TV, BS/TV, and Tb.N and increased Tb.Sp, and overexpression of Sialin failed to restore bone homeostasis (Fig. 5d–h). In cortical bone, Sialin overexpression increased Ct.BV, Ct.Th, and Tt.Ar compared with vector controls, whereas Stattic treatment reduced these parameters (Fig. S6c–e). Sialin overexpression failed to rescue cortical deficits under Stattic treatment (Fig. S6c–e). The level of the bone resorption marker CTX-1 increased by Stattic treatment, and overexpression of Sialin failed to inhibit osteoclast activity and bone resorption status (Fig. S6f). The level of the bone formation marker PINP decreased by Stattic treatment, and overexpression of Sialin failed to enhanced osteoblast activity and bone formation status (Fig. S6g). In addition, hematoxylin and eosin (H&E) and Masson’s trichrome staining revealed structural disruption and decreased collagen content in the femur of the Stattic group, which also could not be restored by the overexpression of Sialin (Fig. 5i, j). IF staining showed that bones with overexpressed Sialin had many Osx^+^ osteoblasts (Fig. 5k, l). Osx^+^ osteoblasts were hardly observed in mouse bones after Stattic treatment, and overexpression of Sialin failed to increase the number (Fig. 5k, l). Quantification of TRAP staining in tibial sections showed that although Sialin inhibited basal osteoclast formation, it failed to suppress the Stattic-induced increase in osteoclast number (Fig. S6h). Taken together, these results suggest that Sialin regulates mitochondrial homeostasis via pSTAT3^S727^, thereby promoting osteogenic differentiation of MSCs and maintaining bone homeostasis.

### Nitrate activates Sialin to restore MSCs' function and prevent age-related bone loss

**Fig. 6:**
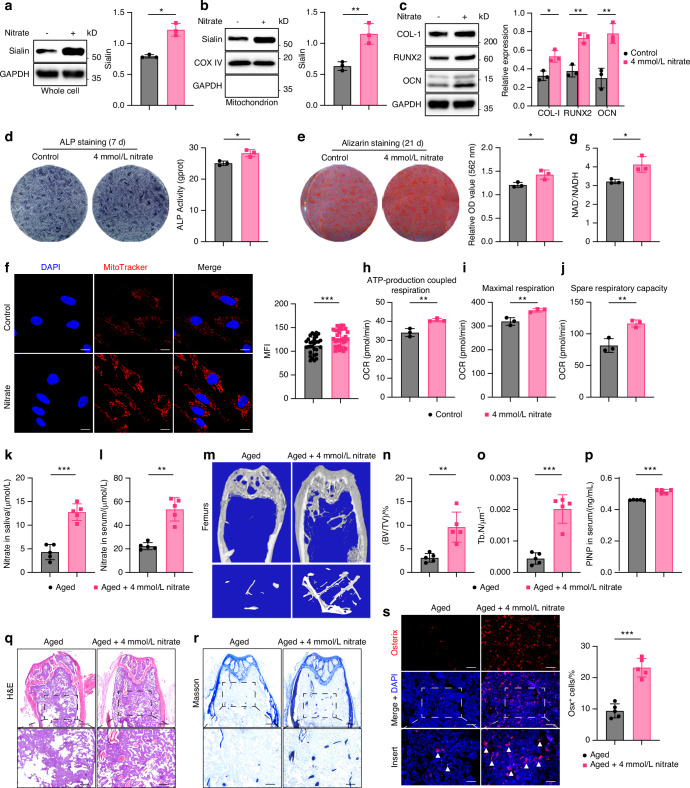
Nitrate-Sialin signaling is closely associated with senile osteoporosis in vivo. **a** Immunoblot analysis of Sialin expression in hMSCs treated with nitrate. **b** Immunoblot analysis of mitochondrial Sialin in hMSCs following nitrate treatment. **c** Immunoblot analysis of COL-I, RUNX2, and OCN in hMSCs treated with nitrate. ALP staining and activity (**d**), and ARS staining and mineralized nodule formation (**e**) in hMSCs treated with nitrate. **f** IF staining images and quantitation of MitoTracker fluorescence intensity in hMSCs treated with nitrate. Scale bars, 25 μm. Quantification of NAD^+^/NADH ratio (**g**), ATP production (**h**), maximal respiration (**i**), and spare respiratory capacity (**j**) in hMSCs following nitrate stimulation. Nitrate concentrations in saliva (**k**) and serum (**l**) of aged mice treated with nitrate. **m** Representative micro-CT images of femurs. Upper scale bars, 1 mm. Lower scale bars, 200 μm. Quantification of BV/TV (**n**) and Tb.N (**o**) in femurs from aged mice treated with nitrate. **p** Serum levels of PINP in aged mice following nitrate treatment. q, r, H&E (**q**) and Masson’s trichrome staining (**r**) in femoral sections from aged mice treated with nitrate. Upper scale bars, 1 mm. Lower scale bars, 200 μm. **s** IF staining images and quantification of Osx in femoral sections from aged mice treated with nitrate. Scale bars: 50 μm. Arrowheads indicate Osx-positive osteoblasts. Data are presented as the mean ± SD, *n* = 3, except for f (*n* = 30), except for **k**, **l**, **n**–**p**, **s** (*n* = 5). *P < 0.05; **P < 0.01; ***P < 0.001

A positive feedback loop between nitrate and Sialin has been proposed to support nitrate-mediated regulation of nitric oxide (NO) homeostasis. In our study, nitrate treatment significantly upregulated Sialin expression in whole hMSCs (Fig. 6a) and specifically within the mitochondria (Fig. 6b), suggesting that nitrate may promote MSCs functional homeostasis via Sialin activation. Functionally, nitrate stimulation enhanced ALP staining intensity in hMSCs, with consistent increases observed in ALP activity (Fig. 6d), and markedly promoted mineralized nodule formation (Fig. 6e). The mRNA expression levels of osteogenic markers, including *ALPL*, *BGLAP*, *SPP1*, and *RUNX2* (Fig. S7a–d), as well as the protein expression of COL-I, RUNX2, and OCN (Fig. 6c), were significantly upregulated following nitrate treatment. Moreover, nitrate treatment increased MMP, as evidenced by elevated MitoTracker fluorescence intensity (Fig. 6f), and improved mitochondrial redox status, as reflected by a higher NAD^+^/NADH ratio (Fig. 6g). OCR analysis further demonstrated that ATP production (Fig. 6h), maximal respiration (Fig. 6i), and spare respiratory capacity (Fig. 6j) were all significantly enhanced in nitrate-treated hMSCs.

To further explore the preventive effect of nitrate–Sialin on age-induced osteoporosis in vivo, we successfully established a model of senile osteoporosis in 18-month-old mice and administered 4 mmol/L nitrate in water for 6 months (Fig. S7e). Nitrate concentrations in the saliva and blood of aged mice significantly increased by nitrate administration (Fig. 6k, l). Micro-CT scanning of the femur microstructure (Fig. 6m) revealed that the BV/TV and Tb.N were elevated while Tb.Sp was decreased after nitrate administration (Fig. 6n, o and Fig. S7f), indicating that nitrate significantly elevated bone volume in aged mice. In aged mice, nitrate administration increased cortical Ct.BV, Ct.Th, and Tt.Ar compared with aged mice, consistent with the trabecular improvements (Fig. S7i–k). The CTX-1 level was significantly reduced in mouse blood after nitrate administration, indicating that nitrate inhibited osteoclast activity and bone resorption status in aged mice (Fig. S7g). The P1NP level was significantly increased in mouse blood after nitrate administration, indicating that nitrate enhanced osteoblast activity and bone formation status in aged mice (Fig. 6p). In addition, H&E and Masson’s trichrome staining revealed improved bone architecture and increased collagen content in aged mice after nitrate administration (Fig. 6q, r). IF staining showed a significant increase in the number of Osx^+^ osteoblasts in the bones of senescent mice (Fig. 6s). Quantitative analysis of TRAP staining in tibial sections showed that nitrate administration significantly reduced osteoclast numbers in aged mice (Fig. S7h). After 21 days of osteogenic induction, ARS staining intensity and mineralized nodule formation were significantly increased in mMSCs isolated from aged mice following nitrate administration (Fig. S7l). In parallel, the mRNA expression levels of *Alpl*, *Spp1*, and *Runx2* were upregulated in these cells (Fig. S7m–o). Taken together, our in vitro and in vivo findings demonstrate that nitrate promotes the osteogenic differentiation of MSCs via Sialin activation, thereby contributing to the attenuation of osteoporosis in aged mice.

## Discussion

Osteoporosis, a systemic metabolic disorder with escalating prevalence in aging populations,^33^ remains inadequately addressed by current therapeutic strategies. The limited efficacy and side effects of existing anti-osteoporosis drugs underscore an urgent need to identify novel targets that regulate bone remodeling through fundamental mechanisms.^34,35^ Our study reveals Sialin (*SLC17A5*), a solute carrier protein traditionally associated with nitrate transport,^18,21^ as a mitochondrial regulator in MSCs that bridges nutrient sensing and osteogenic differentiation. We demonstrate that Sialin localizes to mitochondrial membranes, where it maintains functional homeostasis by recruiting phosphorylated STAT3 at Ser727 (pSTAT3^S727^) to enhance mitochondrial bioenergetics. This mechanism not only expands the known roles of the solute carrier (SLC) family—a vast yet understudied class of membrane transporters^36,37^—but also positions Sialin as a metabolic orchestrator linking environmental nitrate availability to stem cell fate determination. The discovery of its mitochondrial localization challenges the conventional view of SLC proteins as passive nutrient shuttles, instead suggesting their active participation in organelle-specific signaling networks.^14,38^

The functional interplay between Sialin and pSTAT3^S727^ unveils a previously unrecognized layer of mitochondrial regulation. While pSTAT3 has been implicated in mitochondrial respiration through its effects on electron transport chain components,^29,39–43^ our work identifies Sialin as a critical chaperone that facilitates pSTAT3’s mitochondrial translocation and activation. This partnership creates a feedforward loop wherein nitrate uptake through Sialin enhances pSTAT3-dependent oxidative phosphorylation, which in turn amplifies the metabolic capacity required for osteogenic differentiation. Such coordination between a nutrient transporter and transcription factor may represent an evolutionary adaptation to couple environmental nutrient availability with cellular energy demands—a system particularly vital in aging MSCs, where both mitochondrial efficiency and nutrient sensitivity decline. Our findings align with emerging paradigms of metabolism-driven stem cell regulation but introduce a unique twist: rather than merely responding to intracellular metabolites, Sialin actively channels dietary-derived nitrate into a mitochondrial rejuvenation program, effectively turning extracellular nutrients into a differentiation signal.

The therapeutic implications of this nutrient-transporter axis are profound. Dietary nitrate, abundant in leafy vegetables and historically overlooked as a mere metabolic byproduct,^44^ emerges as a potent activator of Sialin-mediated osteogenesis. We show that age-related nitrate depletion correlates with osteopenia, while its restoration through supplementation rescues bone loss in senescent mice by reactivating MSCs' differentiation capacity.^45^ These positions nitrate not as a passive dietary component but as a direct modulator of stem cell metabolism—a concept that bridges nutritional science and regenerative medicine. The self-reinforcing nature of the nitrate-Sialin loop,^18^ where nitrate uptake enhances Sialin expression, creates a therapeutic amplification mechanism with relevance for age-related osteoporosis. Importantly, our work extends beyond previous associations between nitrate deficiency and metabolic syndrome by pinpointing its skeletal-specific effects through MSCs' mitochondrial reprogramming.^46–48^

While these discoveries open new avenues for osteoporosis treatment, several questions remain. The structural basis of Sialin–pSTAT3 interaction requires atomic-level characterization to guide targeted drug design. The pleiotropic effects of Sialin across bone cell types, potentially influencing osteoclast activity or vascular niche formation, demand further investigation to ensure therapeutic specificity. Moreover, translating nitrate supplementation into clinical practice will necessitate rigorous pharmacokinetic studies to optimize dosing regimens and assess long-term safety in elderly populations. Future research should explore whether Sialin polymorphisms correlate with osteoporosis susceptibility, potentially enabling personalized nutrition strategies based on genetic risk profiles. Furthermore, as our in vivo experiments were conducted exclusively in male mice, sex-specific differences in bone metabolism and mitochondrial regulation may have been overlooked. Given the established influence of sex hormones on skeletal energetics and MSC function, future studies should incorporate both sexes to determine whether the nitrate–Sialin–pSTAT3 axis operates similarly in female models.

In conclusion, our study redefines Sialin as a mitochondrial metabolic switch that converts dietary nitrate into a differentiation signal for MSCs. By elucidating the Sialin-pSTAT3 axis as a critical node where environmental nutrients intersect with stem cell metabolism, this work provides a framework for developing “metabologenetic” therapies—interventions that harness nutrient-sensing pathways to counteract age-related tissue degeneration. The integration of nutritional supplementation with transporter-targeted activation represents a promising strategy to rejuvenate MSCs' function, offering a dual approach to osteoporosis management that is both physiologically grounded and therapeutically actionable.

## Materials and Methods

### Mice

This study was approved by the Animal Care and Use Committee of Capital Medical University (AEEI-2023-086). All of the mice were in a C57BL/6 background. *Slc17a5*^−/−^ mice were bred under specific-pathogen-free conditions in the animal facility of the Capital Medical University. *Prrx1-Cre* (*Prx1-Cre*) and *Slc17a5*^*flox/flox*^ (*Slc17a5*^*fl/fl*^) mice were used in the current study. *Prx1-Cre* mice were crossed with *Slc17a5*^*fl/fl*^ mice, and their progeny was bred to obtain MSC-specific conditional knockout mice (*Prx1-Cre*;*Slc17a5*^*fl/fl*^, cKO, 8 weeks). *Slc17a5*^*fl/fl*^ (*flox*) mice served as the control (WT, 8 weeks). All experiments involving cKO and WT mice were conducted using 8-week-old male animals. The genotypes were identified by isolating DNA from the tails of newborn mice and amplifying it by PCR. The sequences of the primers used for genotyping have been listed in Table S1.

Male C57BL/6 mice (8 weeks), purchased from Beijing Vital River Laboratory Animal (Beijing, China), were randomly divided into two groups (*n* = 10/group), one group was injected intraperitoneally with saline (control group), and the other group was injected intraperitoneally with Stattic (10 mg/kg, MedChemExpress, China), 3 times per week for 4 weeks. The control and Stattic groups were further randomly assigned to two groups, adeno-associated virus-9 (AAV9)-Vector and AAV9-HA-*Slc17a5* group (*n* = 5/group). Each mouse received a tail vein injection (i.v.) of AAV9, 5 × 10^11^ vg/mouse, once, and samples were collected after 6 weeks. Male C57BL/6 mice (18 weeks), purchased from Beijing Vital River Laboratory Animal (Beijing, China), were randomly divided into two groups (*n* = 5/group), including the aged group and the 4 mmol/L nitrate group (administration of 4 mmol/L nitrate for 6 months). Inorganic nitrate (Sigma-Aldrich, USA) was dissolved in drinking water for administration. Mice were maintained in a specific pathogen-free animal facility and kept under conventional conditions with free access to water and food.

### Micro-computed tomography (micro-CT) analysis

The femoral bone structure of mice was analyzed using a high-resolution Inveon CT/PET/SPECT scanner (Siemens, Germany). The region of interest (ROI) was defined at the distal femoral metaphysis, starting 0.5 mm proximal to the growth plate and extending proximally for 1.0 mm, excluding the cortical shell. For cortical bone analysis, a 1.0-mm ROI was selected at the mid-diaphysis. Scanning was performed at 80 kV tube voltage, 500 μA tube current, 8.7 μm isotropic voxel size, and 200 ms integration time. Two- and three-dimensional images were reconstructed, and bone morphometric parameters were calculated according to standard guidelines.^49^ Analysis was performed using CT-Analyser, CT-Volume, and CT-Voxel software (Skyscan, Belgium).

### Histological analyses

The femurs of mice were fixed with 4% paraformaldehyde (PFA) for 2 days and then decalcified in 10% EDTA for 2 months. Sections (5-μm) were prepared, stained with hematoxylin and eosin (H&E) and Masson’s trichrome (Maxim Biotechnologies, China) according to the manufacturer’s protocol. The TRAP staining of bone sections was performed as we previously described.^50^

### Cell culture of human MSCs and murine MSCs

Human MSCs were obtained from Procell Life Science & Technology (Wuhan, China). Murine MSCs were cultured from the bone cavity of femurs and tibias of C57BL/6 mice. The cells were cultured with alpha-MEM medium (Gibco, USA) supplemented with 20% fetal bovine serum (FBS, Gibco), 2 mmol/L glutamine, 100 U/mL penicillin, and 100 mg/mL streptomycin (Invitrogen, USA), and then incubated in 5% carbon dioxide at 37 °C. MSCs between passages 3–4 were used in the following study. In this study, 4 mmol/L nitrate (Sigma-Aldrich), 50 nmol/L Rotenone (Rot, Sigma-Aldrich), 30 μmol/L Coenzyme Q10 (CoQ10, Sigma-Aldrich), and 20 μmol/L Stattic were used to stimulate MSCs.

### Plasmid construction and viral infection

According to standard techniques, the plasmids were constructed, and all structures were verified by enzyme digestion and/or sequencing. Human full-length Sialin (*SLC17A5*) complementary DNA (cDNA) was constructed via a whole-gene synthesis method. Next, the *SLC17A5* sequence was fused to an HA-tag sequence (HA-*SLC17A5*) or inserted into the mitochondrial target COX8A (HA-Mito-*SLC17A5*), and subcloned into the GV348 lentiviral vector. The *SLC17A5* sequence was fused to a GFP-tag sequence (GFP-*SLC17A5*), and subcloned into the GV218 lentiviral vector. Short hairpin RNAs (shRNAs) with sequences complementary to those of the target genes were subcloned into the GV493 lentiviral vector. The target sequences for the shRNAs were as follows: *SLC17A5* shRNA (sh-*SLC17A5*), 5′- TGTGAATCTGAGTGTTGCGTTAGTGGATA-3′; Control shRNA (Scramble), 5′- TTCTCCGAACGTGTCACGT-3′. AAV9 vectors were recombined with *Slc17a5* coding sequence. All virus vectors were designed and constructed by GeneChem (Shanghai, China). For viral infections, MSCs were plated overnight and then infected with lentiviruses in the presence of polybrene (6 μg/mL, Sigma-Aldrich) for 24 h. After 48 h, infected cells were selected with 1 μg/mL puromycin (Sigma-Aldrich) for 7 days. In both experiments, western blot analysis of Sialin was performed to assess knockdown or overexpression efficiency.

### Artificial intelligence to predict protein interactions

We utilize a two-view learning model, HIGH-PPI, for predicting protein-protein interactions (PPIs) and extrapolating the molecular details involved, which uses chemical descriptors rather than protein sequences in order to better capture the structure-function relationships of proteins, and has high prediction accuracy and robustness to PPIs, which can explain the PPI model of action by accurately identifying important binding and catalytic sites.^28^

### Co-immunoprecipitation (CoIP) assay

Proteins were incubated with IgG (control), Sialin, or pSTAT3^S727^ conjugated Dynabeads (Life Technologies) at room temperature for 4 h. Following several antibody tests, the rabbit polyclonal anti-Sialin antibody from Thermo and the rabbit monoclonal anti-pSTAT3^S727^ antibody from CST yielded efficient immunoprecipitation results. The supernatant containing unbound material was subsequently removed, and the beads were washed 5 times with low-salt NET-2 buffer (50 mmol/L Tris-HCl, pH 7.4, 150 mmol/L NaCl, 0.05% NP-40, EDTA-free protease inhibitor cocktail (Roche)), or high-salt NET-2 buffer (with 300 mmol/L NaCl), and once with 1× PBS. The bound proteins were eluted using dye-less buffer, precipitated with methanol/chloroform, and detected with the antibodies described above.

### Surface Plasmon Resonance (SPR) analysis

HEK293T cells were used as a mammalian expression system for the transient transfection of Sialin, and the recombinant protein was purified for SPR assays. For bacterial expression, E. coli BL21 (DE3) cells were transformed with pSTAT3^S727^ for protein production. SPR experiments were performed on a Biacore system using CM5 sensor chips. The ligand protein pSTAT3^S727^ was diluted to 20 μg/mL in 10 mmol/L sodium acetate at different pH values to determine the optimal immobilization conditions. After covalent coupling of the ligand to the chip surface, the reference channel was blocked by activation and deactivation steps. Serial dilutions of the analyte (Sialin) were prepared in a 3-fold gradient with a maximum concentration of 1 μmol/L (0.33 μmol/L, 0.11 μmol/L, 0.037 μmol/L, and 0.012 μmol/L). Binding assays were performed with an association time of 120 s, a dissociation time of 80 s, and a flow rate of 30 μL/min.

### Statistical analysis

All experiments are randomized into groups of similar sample size by block randomization. Data collection and analysis were performed blindly. All the experiments were performed independently at least three times, and data were expressed as mean ± SD. Statistical analyses were performed using GraphPad Prism v7.02 (GraphPad). For two-group comparisons of normally distributed data, an unpaired two-sided Student’s *t*-test was applied, with Welch’s correction used when variances were unequal. For multiple group comparisons, an ordinary one-way ANOVA was performed, followed by Tukey’s or Dunnett’s post hoc test as specified in the figure legends. Brown–Forsythe and Welch’s corrections were applied when variances were unequal. A *P* value < 0.05 was considered statistically significant.

## Supplementary information

Supplementary information

## Data Availability

All data supporting the findings of this study are available in the article and its Supplementary Information section. Source data are provided in this paper.
