# Supplementary material for: Sialin-STAT3 axis regulates bone homeostasis in mice
Source: Bone Res. 2026 Feb 9;14:20. doi: 10.1038/s41413-025-00504-2 (PMC12887030; doi:10.1038/s41413-025-00504-2)
Supplement: Supplementary file 1 — Supplementary information [file 41413_2025_504_MOESM1_ESM.pdf]

Supplementary information for:

Sialin-STAT3 axis regulates bone homeostasis in mice

Xiaoyu Li[1], Lei Hu[1], Yifan Xu[1], Xue Wang[1], Zichen Cao[1], Ou Jiang[1], Jiawei Yao[4], Meijing Liu[4], Sihan Kong[1], Jinsong Wang[1,2], Xiaogang Wang[4*], and Songlin Wang[1,2,3*]

Correspondence: slwang@ccmu.edu.cn (S.W.) & xiaogangwang@smu.edu.cn (X.W.)

This file include:

Supporting Information of Materials and Methods

Figures and legends S1 to S7

Table S1

Supporting Information of Materials and Methods

Nitrate level determination

Saliva and serum of mice from each group were filtered and diluted by 10 000 MW. The concentration of nitrate was detected with Total Nitric Oxide and Nitrate/Nitrite Parameter Assay Kit (R&D, USA), following standard experimental procedures provided by the manufacturer.

Enzyme-linked immunosorbent (ELISA) assay

The level of the bone formation marker procollagen type I N-terminal propeptide (PINP) and the bone resorption marker collagen type I cross-linked C-telopeptide (CTX-1) in the serum of mice from each group were measured by ELISA kits (Elabscience, China) according to the manufacturer's instructions.

Osteogenic differentiation detection

MSCs were cultured with osteogenic inductive medium (Cyagen, China). After 7 days, MSCs were stained with ALP Color Development Kit (Beyotime Biotechnology). ALP activity level was measured using the ALP activity assay kit (Nanjing Jiancheng Bioengineering Institute) and measured at a wavelength of 520 nm with a microplate reader. After 21 days, the mineralized nodules were stained with 2% Alizarin Red (Sigma-Aldrich). After solubilizing in 10% cetylpyridinium chloride (CPC, Sigma-Aldrich) for 30 minutes (min) at room temperature, areas of the Alizarin red stain were measured by the microplate reader at a wavelength of 560 nm.

Mitochondrial activity

MSCs were incubated in alpha-MEM medium (Gibco) at 37 °C with (a) Mito-Tracker Deep Red (Invitrogen) 200 nM for 30 min, (b) JC-1 (Sigma) 10 ug/ml for 30 min, as per the manufacturer’s instructions. Cells were then washed with PBS and analyzed immediately.

NAD^+/NADH quantification

MSCs were collected into microcentrifuge tubes by centrifugation at 2 000 rpm for 5 min. Cells were extracted with 400 μl of NADH/NAD Extraction Buffer by homogenization or by two freeze–thaw cycles (20 min on dry ice followed by 10 min at room temperature). The lysates were centrifuged at 13 000 *g* for 10 min to remove insoluble material. The supernatants were assayed for NAD^+/NADH content according to the manufacturer's instructions, and absorbance was measured at 450 nm.

ATP determination

ATP was determined using ATP assay kit (Sigma). Lyse MSCs in 100 ul of ATP Assay Buffer. ATP concentration is determined by phosphorylating glycerol, resulting in a colorimetric (570 nm) product proportional to the amount of ATP present.

Cellular ROS Detection Assay

We analyzed the cellular ROS using the fluorometric intracellular reactive oxygen species kit (Sigma-Aldrich) according to the manufacturer's instructions. Briefly, cells were incubated with ROS detection reagent stock solution for 30 min in the dark at 37 °C in 5% CO_2. Subsequently, the fluorescence intensity was measured by flow cytometer with an APC filter.

Oxygen consumption rate (OCR) and extracellular acidification rate (ECAR) analysis

OCR and ECAR were measured using a seahorse XF24 analyzer (Seahorse bioscience). For OCR measurement, cells were covered with 500 μl assay medium (XF base medium, 1 mM sodium pyruvate, 1 mM L-glutamine and 10 mM glucose). Port injections were performed with 1 mM oligomycin, 3 mM FCCP, 0.5 mM antimycin and rotenone. For ECAR measurement, cells were covered with 500 μl assay medium (XF base medium, 1 mM L-glutamine). Port injections were performed with 10 mM glucose, 1 mM oligomycin and 50 mM 2-DG.

Immunofluorescence staining

MSCs were fixed with 4% PFA for 15 min and permeabilized with 0.5% Triton X-100 for 20 min at room temperature. Then cells were blocked with normal goat serum for 30 min, and finally incubated with rabbit polyclonal anti-Sialin (Thermo Scientific, USA), mouse monoclonal anti-HA (CST, USA), rabbit monoclonal anti-TOMM20 (CST), rabbit monoclonal anti-pSTAT3^{S727} (CST) and rabbit monoclonal anti-Osterix (CST) overnight at 4 °C. The next day, cells were incubated with donkey anti-mouse IgG (H+L) Alexa Fluor 488 (Invitrogen, USA) and donkey anti-rabbit IgG (H+L) Alexa Fluor 488/594 (Invitrogen) for one hour at room temperature before counterstaining the nuclei with DAPI (Invitrogen). IF images were taken using the confocal microscopy (Leica, Germany).

Single-molecule imaging

Single-molecule structured illumination microscopy (SIM) was performed using the DEEPSIM super-resolution imaging microscope (CrestOptics, Italy). MSCs were fixed, permeabilized, and immunostained with antibodies against Sialin and mitochondrial markers (TOMM20), followed by fluorescent secondary antibodies. Images were acquired with a maximum XY resolution of 100 nm and Z resolution of 300 nm, with a step size of 0.125 μm.

RT-PCR analysis

Total RNA was isolated from MSCs using the RNAios Plus reagent (TaKaRa, Japan) and was reverse transcribed to cDNA using the PrimeScript TM RT Reagent Kit with gDNA Eraser (TaKaRa). Target genes were amplified with cDNA, specific primers and NovoStart®SYBR qPCR SuperMix plus (Novoprotein, China) in a CFX96 Touch Real-Time PCR detection system (Bio-Rad Laboratories, USA). The relative expression level of the target gene was normalized to GAPDH level and determined by the 2−ΔΔCt method. The information on the primers is shown in Table S1.

Mitochondria isolation and western blot analysis

Cellular mitochondria isolation was performed using the Mitochondria Isolation Kit (Thermo) according to the manufacturer's instructions and validated purity by western blot using anti-COX-IV antibody as mitochondria marker. MSCs were lysed using RIPA reagent containing 1% PMSF and 1% phosphatase inhibitor cocktail. After centrifugation at 14 000 *g* for 5 min, total protein concentrations were measured using a BCA Protein Assay Kit (Solarbio). Proteins were then separated by sodium dodecyl sulphate–polyacrylamide gel electrophoresis (SDS-PAGE) and transferred onto polyvinylidene difluoride (PVDF) membranes (Millipore, USA). The protein bands were then developed with the use of the Pierce ECL Western Blotting Substrate (Thermo Fisher Scientific, USA), and the densitometry of each band was conducted using ImageJ (National Institutes of Health). The following primary antibodies were used: COL-I (CST), RUNX2 (CST), BGLAP (Abclonal), SIALIN (Thermo), COX IV (CST), $pSTAT3^{S727}$ (CST), GAPDH (Abcam).

Proteomics analysis

Peptide mixtures were first incubated with IMAC microspheres suspension with vibration in loading buffer (50% acetonitrile/0.5% acetic acid). To remove the non- specifically adsorbed peptides, the IMAC microspheres were washed with 50% acetonitrile/0.5% acetic acid and 30% acetonitrile/0.1% trifluoroacetic acid, sequentially. To elute the enriched phosphopeptides, the elution buffer containing 10% NH_4OH was added and the enriched phosphopeptides were eluted with vibration. The supernatant containing phosphopeptides was collected and lyophilized for LC-MS/MS analysis. The tryptic peptides were dissolved in solvent A (0.1% formic acid, 2% acetonitrile/in water), directly loaded onto a home-made reversed-phase analytical column (25 cm length, 75/100 μm i.d.). Peptides were separated with a gradient from 6% to 24% solvent B (0.1% formic acid in acetonitrile) over 70 min, 24% to 35% in 14 min and climbing to 80% in 3 min then holding at 80% for the last 3 min, all at a constant flow rate of 450 nl/min

on a nanoElute UHPLC system (Bruker Daltonics). The peptides were subjected to capillary source followed by the timsTOF Pro (Bruker Daltonics) mass spectrometry. The resulting MS/MS data were processed using MaxQuant search engine (v.1.6.15.0). Tandem mass spectra were searched against the human SwissProt database (20422 entries) concatenated with reverse decoy database. FDR was adjusted to < 1%. Proteins with a P value < 0.05 or fold change > 2 were identified as differentially expressed. Subsequently, GO functional annotation analysis, Reactome pathway analysis, and WikiPathway analysis of the differentially expressed proteins were performed.

Supporting Figures and Figure Legends

Figure S1

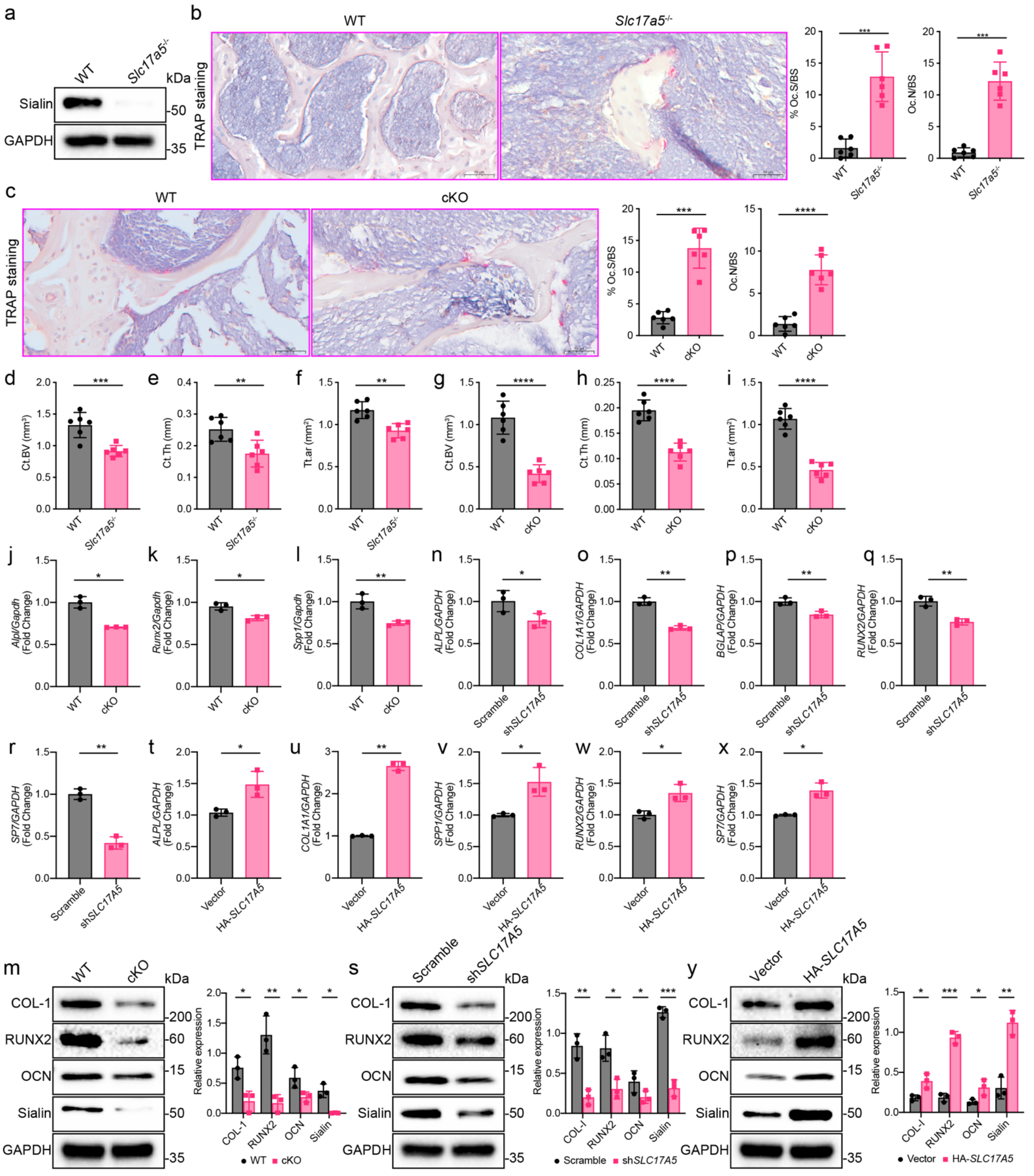

Figure S1 Expression of osteogenesis-related genes in *SLC17A5* knockdown or overexpression MSCs.

a, Immunoblot analysis of Sialin in mMSCs from WT and *Slc17a5*$^{-/-}$ mice. b, c, Tartrate-resistant acid phosphatase (TRAP) staining and quantification of femoral sections from *Slc17a5*$^{-/-}$ (b) and cKO mice (c). Scale bars, 50μm. d–f, Quantification of cortical bone volume (Ct.BV, d), cortical thickness (Ct.Th, e), and total trabecular area (Tt.Ar, f) in WT and *Slc17a5*$^{-/-}$ mice. g–i, Quantification of cortical bone volume (Ct.BV, g), cortical thickness (Ct.Th, h), and total trabecular area (Tt.Ar, i) in WT and cKO mice. j–l, RT-qPCR analysis of *Alpl*, *Runx2*, and *Spp1* in mMSCs from 8-week-old WT and cKO male mice. m, Immunoblot analysis of COL-I, RUNX2, and OCN in mMSCs from 8-week-old WT and cKO male mice. n–r, RT-qPCR analysis of *ALPL*, *COL1A1*, *BGLAP*, *RUNX2*, and *SP7* in control and Sialin knockdown (sh*SLC17A5*) hMSCs. s, Immunoblot analysis of COL-I, RUNX2, Sialin, and OCN in control and sh*SLC17A5* hMSCs. t–x, RT-qPCR analysis of *ALPL*, *COL1A1*, *SPP1*, *RUNX2*, and *SP7* in control and Sialin-overexpressing (HA-*SLC17A5*) hMSCs. y, Immunoblot analysis of COL-I, RUNX2, Sialin, and OCN in control and Sialin-overexpressing hMSCs. Data are presented as the mean ± SD, n=3, except for b–i (n=6). $^{*}P<0.05$; $^{**}P<0.01$.

Figure S2

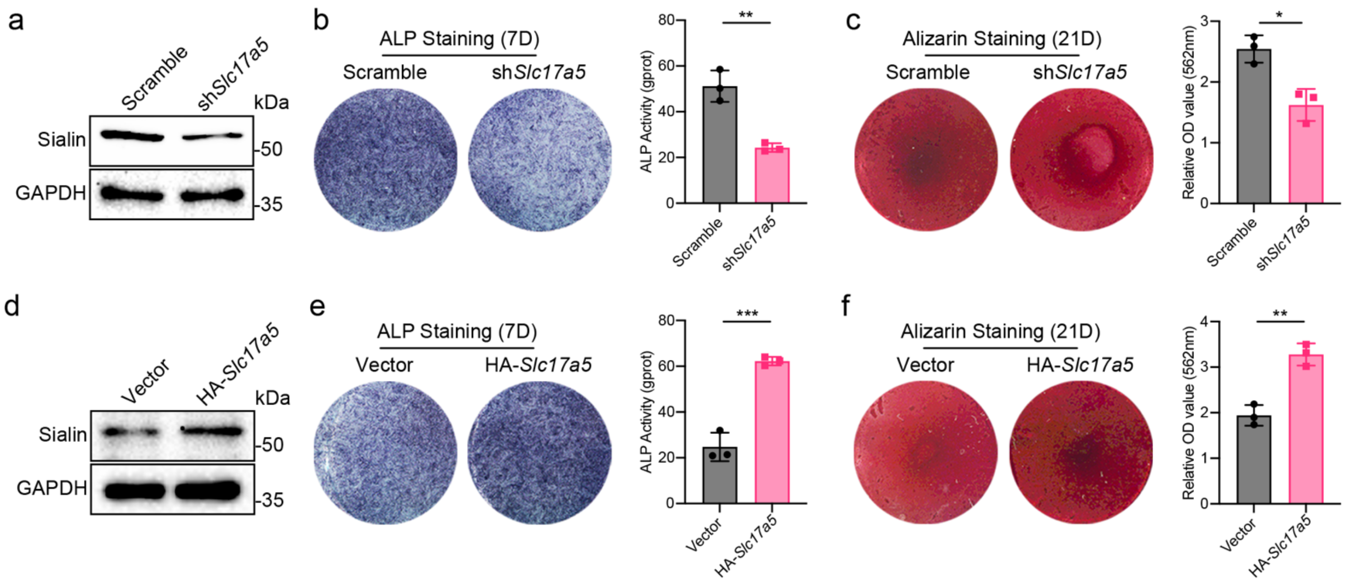

Figure S2 Regulation of osteogenic differentiation in *Slc17a5* knockdown or overexpressing murine MSCs. a, Immunoblot analysis of Sialin expression in control and sh*Slc17a5* mMSCs from 8-week-old male C57BL/6 mice. b, c, ALP staining and activity (b), and ARS staining with quantification of mineralized nodule formation (c) in control and sh*Slc17a5* mMSCs from 8-week-old male C57BL/6 mice. d, Immunoblot analysis of Sialin expression in control and Sialin-overexpressing (HA-*Slc17a5*) mMSCs from 8-week-old male C57BL/6 mice. e, f, ALP staining and activity (e), and ARS staining and mineralization (f) in control and Sialin-overexpressing mMSCs from 8-week-old male C57BL/6 mice. Data are presented as the mean ± SD, n=3. *P<0.05; **P<0.01; ***P<0.001.

Figure S3

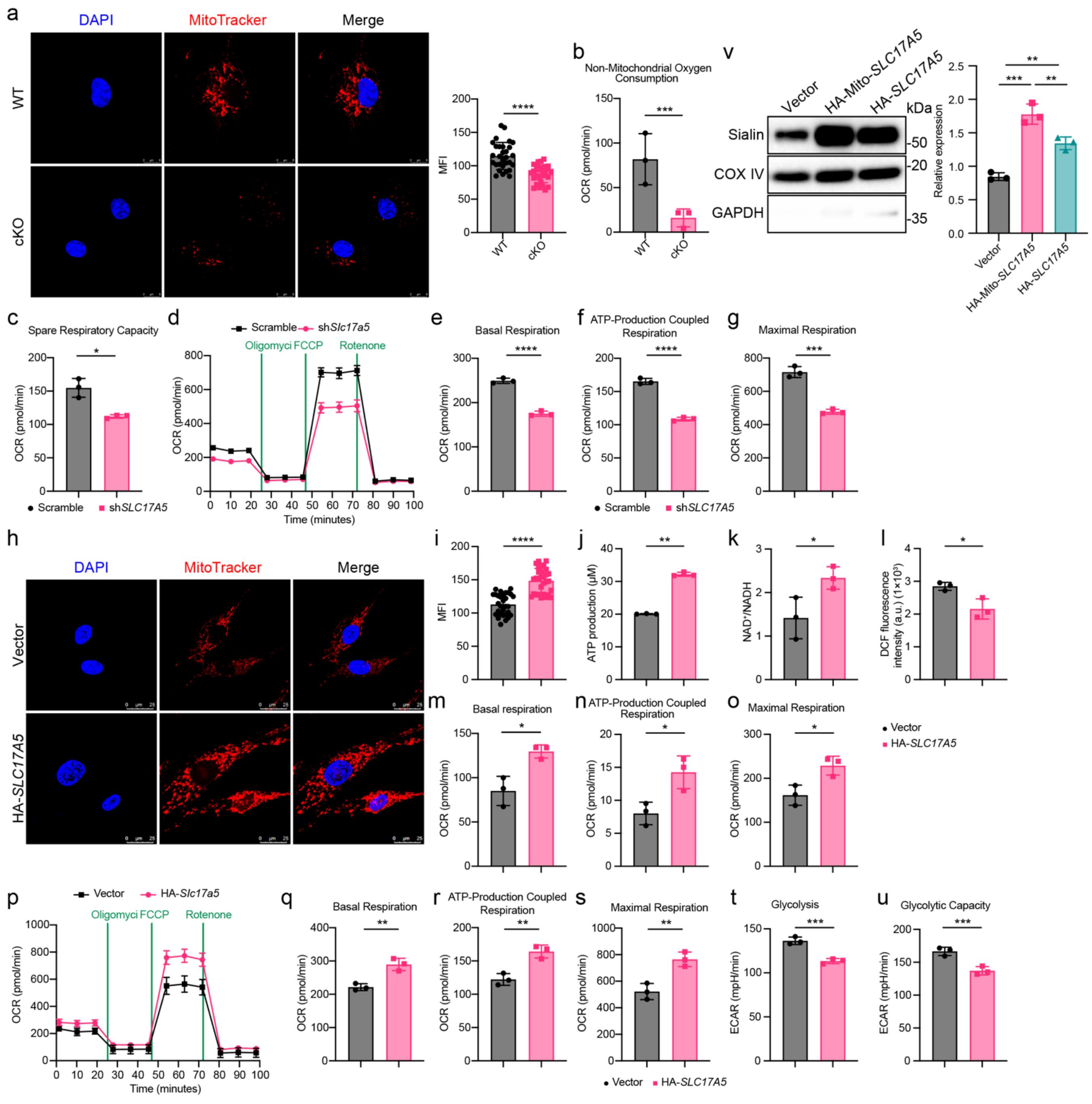

Figure S3 Sialin regulates mitochondrial function in human and murine MSCs. a, IF staining images and quantitation of MitoTracker intensity in mMSCs from 8-week-old male WT and cKO mice. Scale bars, 25 μm. b, Non-mitochondrial oxygen consumption in mMSCs from 8-week-old male WT and cKO mice. c, Spare respiratory capacity in control and sh*SLC17A5* hMSCs. d, OCR measurement in control and sh*Slc17a5* mMSCs from 8-week-old male C57BL/6 mice. e–g, Quantification of basal respiration (e), ATP production (f), and maximal respiration (g) in control and sh*Slc17a5* mMSCs from 8-week-old male

C57BL/6 mice. h, i, IF staining images (h) and quantitation (i) of MitoTracker fluorescence in control and Sialin-overexpressing hMSCs. Scale bars, 25 μm. j–o, ATP level (j), NAD^+/NADH ratio (k), ROS level (l), basal respiration (m), ATP production (n), and maximal respiration (o) in control and Sialin-overexpressing hMSCs. p, OCR measurement in control and Sialin-overexpressing (HA-*Slc17a5*) mMSCs from 8-week-old male C57BL/6 mice. q–s, Quantification of basal respiration (q), ATP production (r), and maximal respiration (s) in control and Sialin-overexpressing mMSCs from 8-week-old male C57BL/6 mice. t, u, Glycolysis (t), and glycolytic capacity (u) in control and Sialin-overexpressing (HA-*SLC17A5*) hMSCs. v, Immunoblot analysis of Sialin expression in mitochondrial fractions from control, Sialin-overexpressing (HA-*SLC17A5*), and mitochondria-targeted Sialin-overexpressing (HA-Mito-*SLC17A5*) hMSCs. Data are presented as the mean ± SD, n=3, except for a (n=30). *P<0.05; **P<0.01; ***P<0.001; ****P<0.0001.

Figure S4

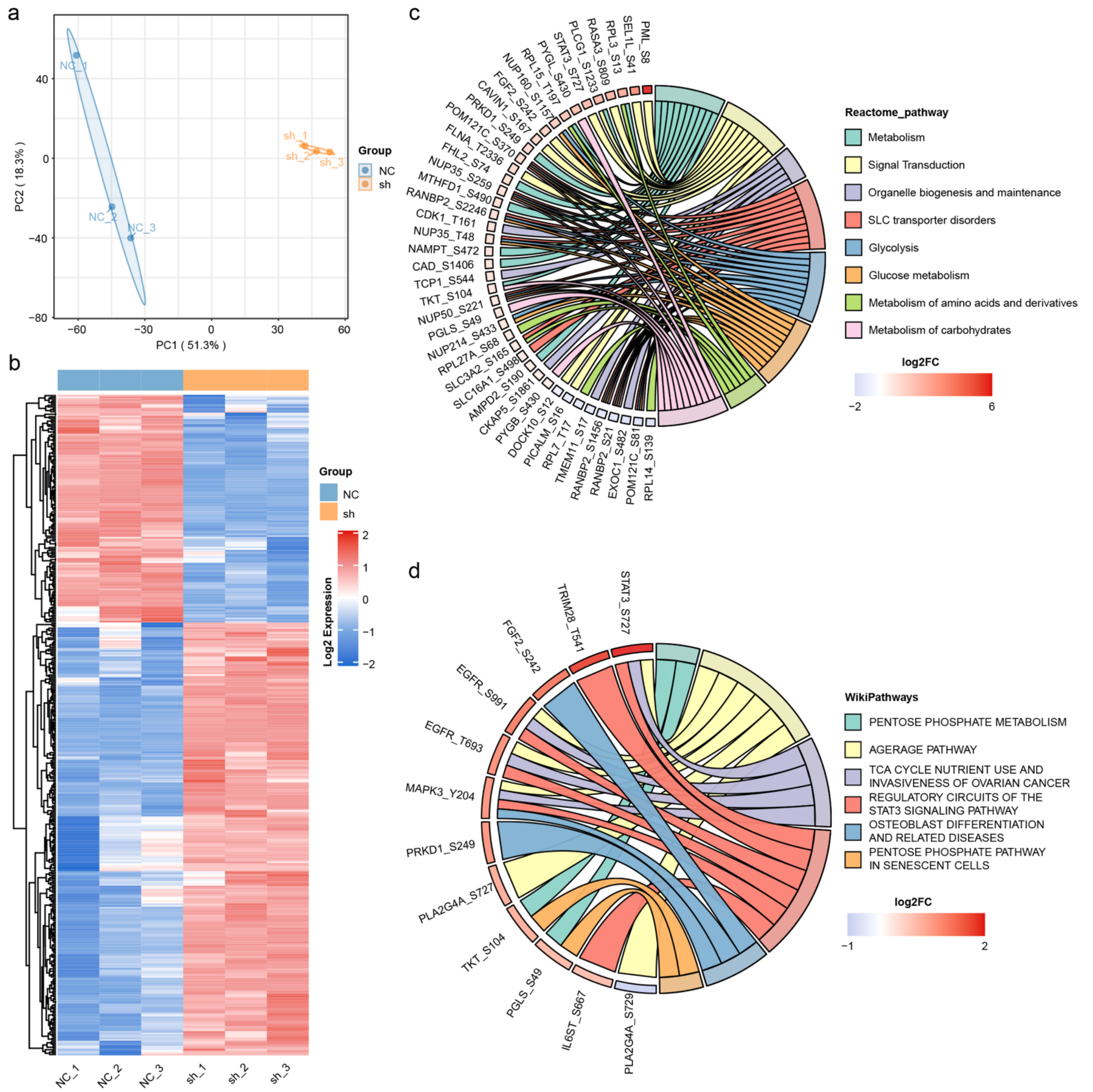

Figure S4 Phosphoproteomic profiling of mitochondrial proteins in control and *SLC17A5* knockout hMSCs. a, Principal component analysis (PCA) of mitochondrial phosphoproteome. b, Heatmap showing differentially phosphorylated mitochondrial proteins. c, Reactome pathway enrichment analysis of differentially phosphorylated proteins. d, Enrichment analysis of differentially phosphorylated proteins using WikiPathways. n=3.

Figure S5

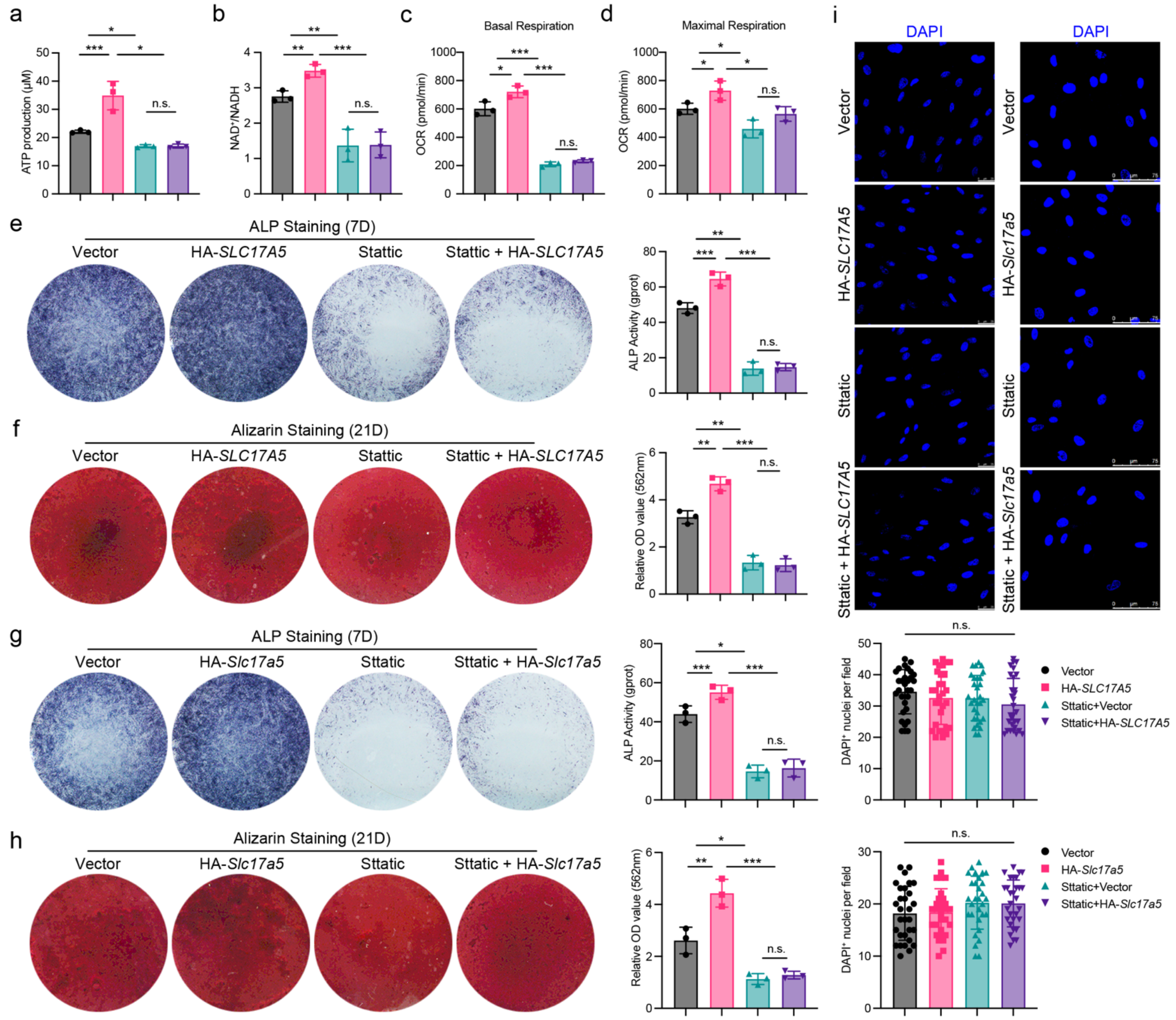

Figure S5 Inhibition of STAT3 phosphorylation abolishes the functional enhancement of MSCs induced by Sialin overexpression. a, b, Quantification of ATP level (a) and NAD$^+$/NADH ratio (b) in control and Sialin-overexpressing hMSCs after Stattic treatment. c, d, Basal respiration (c) and maximal respiration (d) in control and Sialin-overexpressing (HA-*Slc17a5*) mMSCs from 8-week-old male C57BL/6 mice following Stattic treatment. e, f, ALP staining and activity (e) and ARS staining and mineralized nodule formation (f) in control and Sialin-overexpressing hMSCs treated with Stattic. g, h, ALP staining and activity (g) and ARS staining with quantification of mineralized nodules (h) in control and Sialin-

overexpressing mMSCs from 8-week-old male C57BL/6 mice treated with Stattic. i, Quantification of total cell number per field by DAPI staining in control and Sialin-overexpressing hMSCs and mMSCs treated with Stattic. Scale bars, 25 μm (upper) and 75 μm (lower). Data are presented as the mean ± SD, n=3. *P<0.05; **P<0.01; ***P<0.001; n.s., no significance.

Figure S6

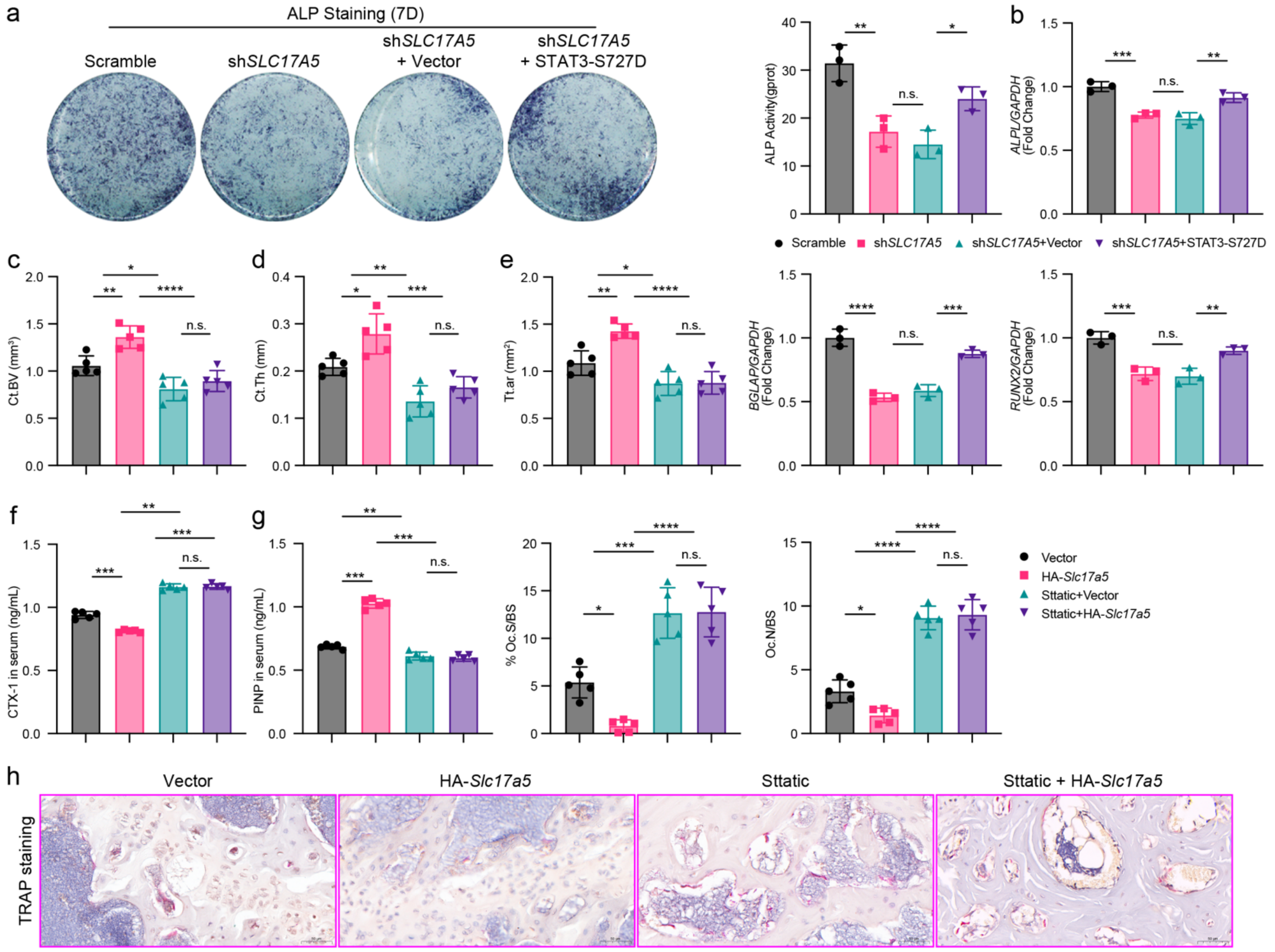

Figure S6 STAT3 phosphorylation mediates the osteogenic effects of Sialin *in vitro* and *in vivo*. a, ALP staining and activity in control and sh*SLC17A5* hMSCs transfected with phosphomimetic STAT3-S727D. b, RT-qPCR analysis of *ALPL*, *BGLAP*, and *RUNX2* mRNA levels in control and sh*SLC17A5* hMSCs transfected with STAT3-S727D. c–e, Quantification of cortical bone volume (Ct.BV, c), cortical thickness (Ct.Th, d), and total trabecular area (Tt.Ar, e) in control and Sialin-overexpressing (HA-*Slc17a5*) mice treated with Stattic. f, g, Serum levels of CTX-1 (f) and PINP (g) in control and Sialin-overexpressing (HA-*Slc17a5*) mice treated with Stattic. h, TRAP staining and quantification of femoral sections from control and Sialin-overexpressing (HA-*Slc17a5*) mice treated with Stattic. Scale bars, 50 μm. Data are presented

as the mean ± SD, n=3, except for c–h (n=5). *$P<0.05$; **$P<0.01$; ***$P<0.001$; ****$P<0.0001$; n.s., no significance.

Figure S7

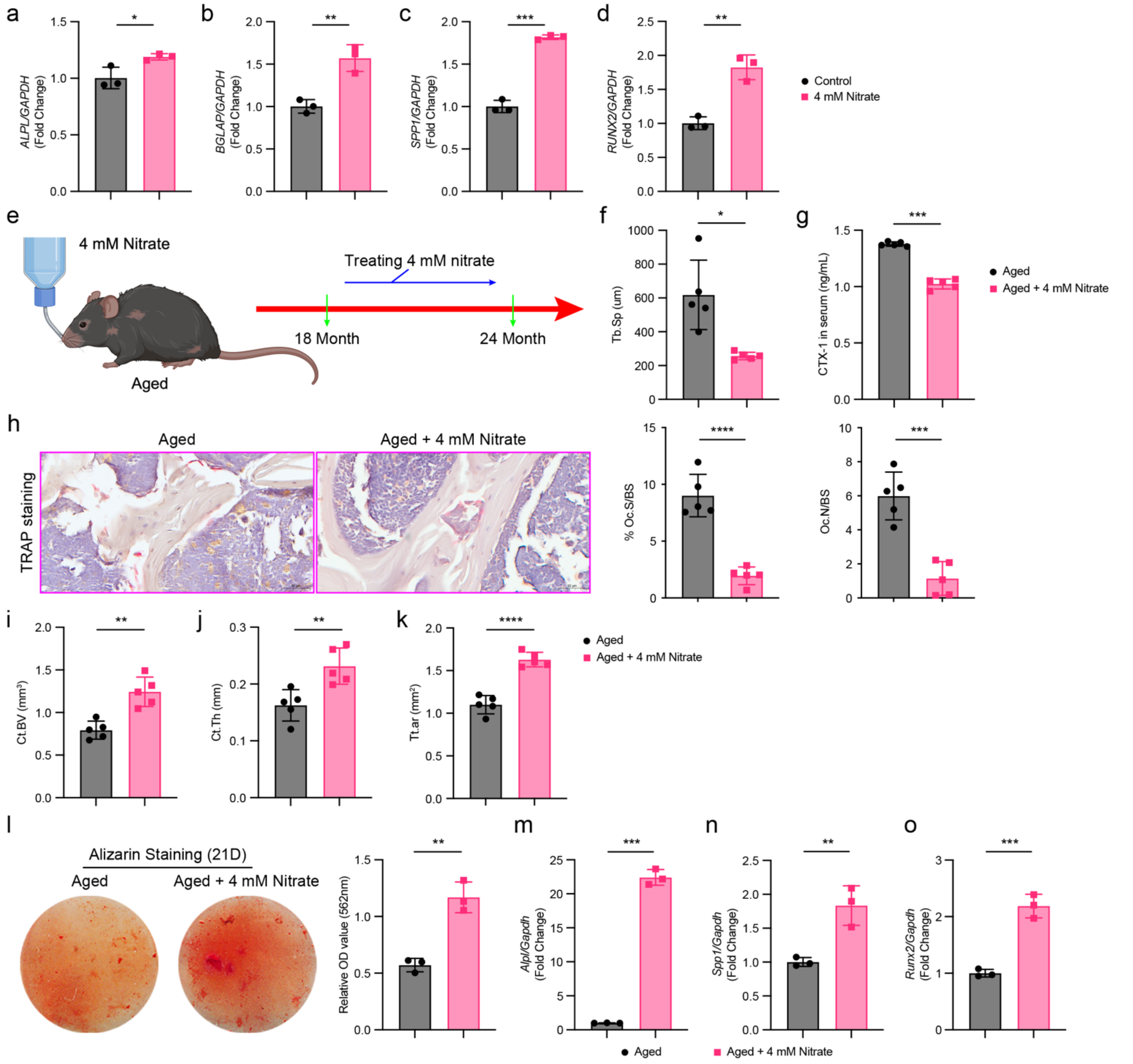

Figure S7 Nitrate enhances osteogenic differentiation in human and murine MSCs. a–d, RT-qPCR analysis of *ALPL*, *BGLAP*, *SPP1*, and *RUNX2* mRNA expression in hMSCs following nitrate treatment. e, Schematic diagram. f, Quantification of Tb.Sp in femurs from aged mice treated with nitrate. g, Serum CTX-1 levels in aged mice treated with nitrate. h, TRAP staining and quantification of femoral sections from aged mice treated with nitrate. Scale bars, 50μm. i–k, Quantification of cortical bone volume (Ct.BV, i), cortical thickness (Ct.Th, j), and total trabecular area (Tt.Ar, k) in aged mice treated with nitrate. l, ARS

staining and quantification of mineralized nodule formation in mMSCs from 18-week-old male C57BL/6 mice treated with nitrate. m–o, RT-qPCR analysis of *Alpl*, *Spp1*, and *Runx2* mRNA expression in mMSCs from 18-week-old male C57BL/6 mice treated with nitrate. Data are presented as the mean ± SD, n=3, except for f–k (n=5). *$P<0.05$; **$P<0.01$; ***$P<0.001$.

Supplementary Table

Table S1. Gene primers

Name	Forward/ Reverse	Sequences
Prx1-Cre	F	5'-GCTCTGATGTTGGCAAAGGGGT-3'
	R	5'-AACATCTTCAGGTTCTGCGGG-3'
Slc17a5-flox-P1	F	5'-CTCCACAGTTCCTCCCATAGAAC-3'
Slc17a5-flox-P2	R	5'-ACAAGCTTGCTAGAAGGTAGGAGA-3'
Slc17a5-flox-P3	F	5'-TTCAGAAGTCAGTTCCCCTCATCA-3'
Slc17a5-flox-P4	R	5'-TATCATGTGAGCTAGATGTGGGTCT-3'
mGapdh	F	5'-AGGAGAGTGTTTCCTCGTCC-3'
	R	5'-TGCCGTGAGTGGAGTCATAC-3'
mAlpl	F	5'-GGTCACAGCAGTTGGTAGCTT-3'
	R	5'-AATTGACGTTCCGATCCTGAGTG-3'
mRunx2	F	5'-TCGGAGAGGTACCAGATGGG-3'
	R	5'-AGGTGAAACTCTTGCCTCGT-3'
mSpp1	F	5'-AAGCATCCTTGCTTGGGTTTG-3'
	R	5'-ATGGTCGTAGTTAGTCCCTCAGA-3'
hGAPDH	F	5'-GGAGCGAGATCCCTCCAAAAT-3'
	R	5'-GGCTGTTGTCATACTTCTCATGG-3'
hALPL	F	5'-TCCTGGGAGATGACGTACAA-3'
	R	5'-AGATTTCCCAGCGTCCTTGG-3'
hCOL-I	F	5'-GCTGATGATGCCAATGTGGTT-3'
	R	5'-CCAGTCAGAGTGGCACATCTTG-3'
hBGLAP	F	5'-AATCCGGACTGTGACGAGTTG-3'
	R	5'-CAGCAGAGCGACACCCTAGAC-3'
hSPP1	F	5'-ATGATGGCCGAGGTGATAGT-3'
	R	5'-ACCATTCAACTCCTCGCTTT-3'
hRUNX2	F	5'-GTTTCACCTTGACCATAACCGT-3'
	R	5'-GGGACACCTACTCTCATACTGG-3'
hSP7	F	5'-TAGGACTGTAGGACCGGAGC-3'
	R	5'-CATAGTGAACTTCCTCCTGGGG-3'
